# Photoinduced Current Transient Spectroscopy on Metal Halide Perovskites: Electron Trapping and Ion Drift

**DOI:** 10.1021/acsenergylett.3c01429

**Published:** 2023-09-26

**Authors:** Giovanni Armaroli, Lorenzo Maserati, Andrea Ciavatti, Pierpaolo Vecchi, Alberto Piccioni, Martina Foschi, Valentina Van der Meer, Chiara Cortese, Matias Feldman, Vito Foderà, Thibault Lemercier, Julien Zaccaro, Javier Mayén Guillén, Eric Gros-Daillon, Beatrice Fraboni, Daniela Cavalcoli

**Affiliations:** # Department of Physics and Astronomy, University of Bologna, 40127 Bologna, Italy; § University Grenoble Alpes, CNRS, Grenoble INP, Institut Néel, F38042 Grenoble, France; ∥ University Grenoble Alpes, CEA, Liten, F-38000 Grenoble, France; ‡ University Grenoble Alpes, CEA, Leti, F-38000 Grenoble, France

## Abstract

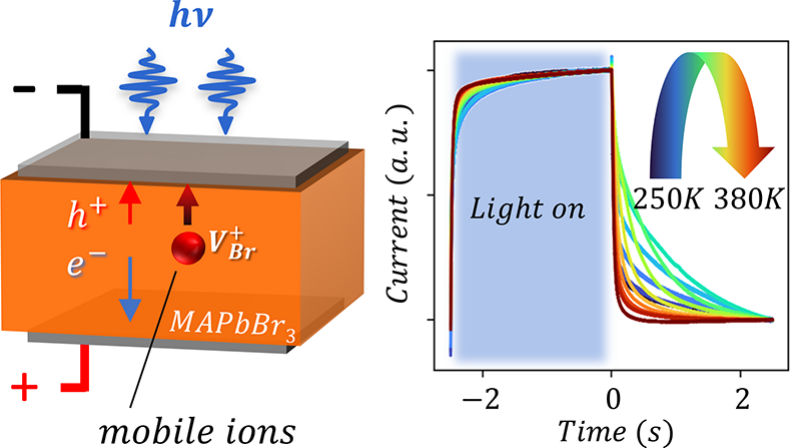

Metal halide perovskites (MHPs) are disruptive materials for a vast class of optoelectronic devices. The presence of electronic trap states has been a tough challenge in terms of characterization and thus mitigation. Many attempts based on electronic spectroscopies have been tested, but due to the mixed electronic–ionic nature of MHP conductivity, many experimental results retain a large ambiguity in resolving electronic and ionic charge contributions. Here we adapt a method, previously used in highly resistive inorganic semiconductors, called photoinduced current transient spectroscopy (PICTS) on lead bromide 2D-like ((PEA)_2_PbBr_4_) and standard “3D” (MAPbBr_3_) MHP single crystals. We present two conceptually different outcomes of the PICTS measurements, distinguishing the different electronic and ionic contributions to the photocurrents based on the different ion drift of the two materials. Our experiments unveil deep level trap states on the 2D, “ion-frozen” (PEA)_2_PbBr_4_ and set new boundaries for the applicability of PICTS on 3D MHPs.

In the past decade, metal halide perovskites (MHPs) have become pervasive in material science research spanning from inorganic chemistry to solid state and device physics. The reasons lie in the excellent performance of MHPs used as low-temperature solution-processable semiconductors for a large variety of optoelectronic applications. In particular, high-energy detection is a very promising application where lead-based bromine perovskites are among the top materials candidates.^1^ The understanding and subsequent mitigation of the electronic trap states that currently limit the charge carrier transport and influence the material environmental stability is pivotal for MHPs’ successful development.^2,3^ To untangle the complex phenomena affecting the optoelectronic response of MHPs to illumination and applied voltage, direct observations under working conditions are highly desirable.

Here we employ a parent technique to deep level transient spectroscopy (DLTS),^4−10^ called photoinduced current transient measurements (PICTS),^11−14^ on 2D-like and 3D lead bromide perovskite single crystals: (PEA)_2_PbBr_4_ and MAPbBr_3_, respectively. PICTS can probe semiconductors through photocurrent variation, rather than capacitance, and it is thus generally suitable to investigate in-gap electronic states in highly resistive materials. Indeed, we show that PICTS can be used to detect (PEA)_2_PbBr_4_ electronic defect states because of the highly reduced ion mobility compared with the 3D MHPs. In contrast, we demonstrate that the photocurrents transient in 3D MHPs such as MAPbBr_3_ are heavily modulated by the ion movement taking place under illumination. We therefore introduce a model for interpreting the PICTS signals in the 3D MHPs, linking the ionic motion to the photocurrent modulation, thus challenging the current understanding of this technique applied to mixed ionic–electronic conductors.^13^

Finally, we interpret the PICTS signal as direct visualization of ionic motion carrying information on the activation energy and diffusivity of the mobile ions, hence ultimately retrieving valuable information on the metal halide perovskite ion mobility via light-driven perturbation under working conditions.

The experimental setup (Figure 1a) allowed us to control the temperature, shine an intermittent above bandgap light, and record photocurrent transients from metal contacted (PEA)_2_PbBr_4_ and MAPbBr_3_ single crystals (Figure 1b,c, respectively; see Figure S1 and Experimental Section for details). The PICTS signals (Figure 1d,e) were derived from the photocurrent dynamics by sampling the current variations at progressive time intervals, after the light is turned off, and replotting these variation versus temperature (see Figure S2 and Supporting Information Discussion for details). In the classic picture, PICTS gives access to trap state properties via the thermally released photogenerated trapped carriers.^11,12^ We argue that this interpretation is valid if the ions are stationary, but it breaks down when ions are sufficiently mobile and their migration governs the photocurrent transients. The ionic contributions to the electrical currents have been largely discussed for MHPs, although the extent of ionic versus electronic current ratio and their relationships are still matters of debate.^14−16^ Ions can modulate the photogenerated currents in direct or in indirect ways: direct if ions are charge carriers responsible for injecting the charges into the metal contacts, indirect if otherwise. In the former case, an ionic current can be modulated either by a direct photoionization (I) or by trapping photogenerated charge carriers with subsequent ion drift (photochemical ionization) (II). In our case, since visible photons are used, direct photoionization cannot be involved. Alternatively, ions can indirectly modulate photogenerated charge carriers by controlling their recombination rates (III)^17,18^ or dynamic doping (IV).^19,20^

**Figure 1:**
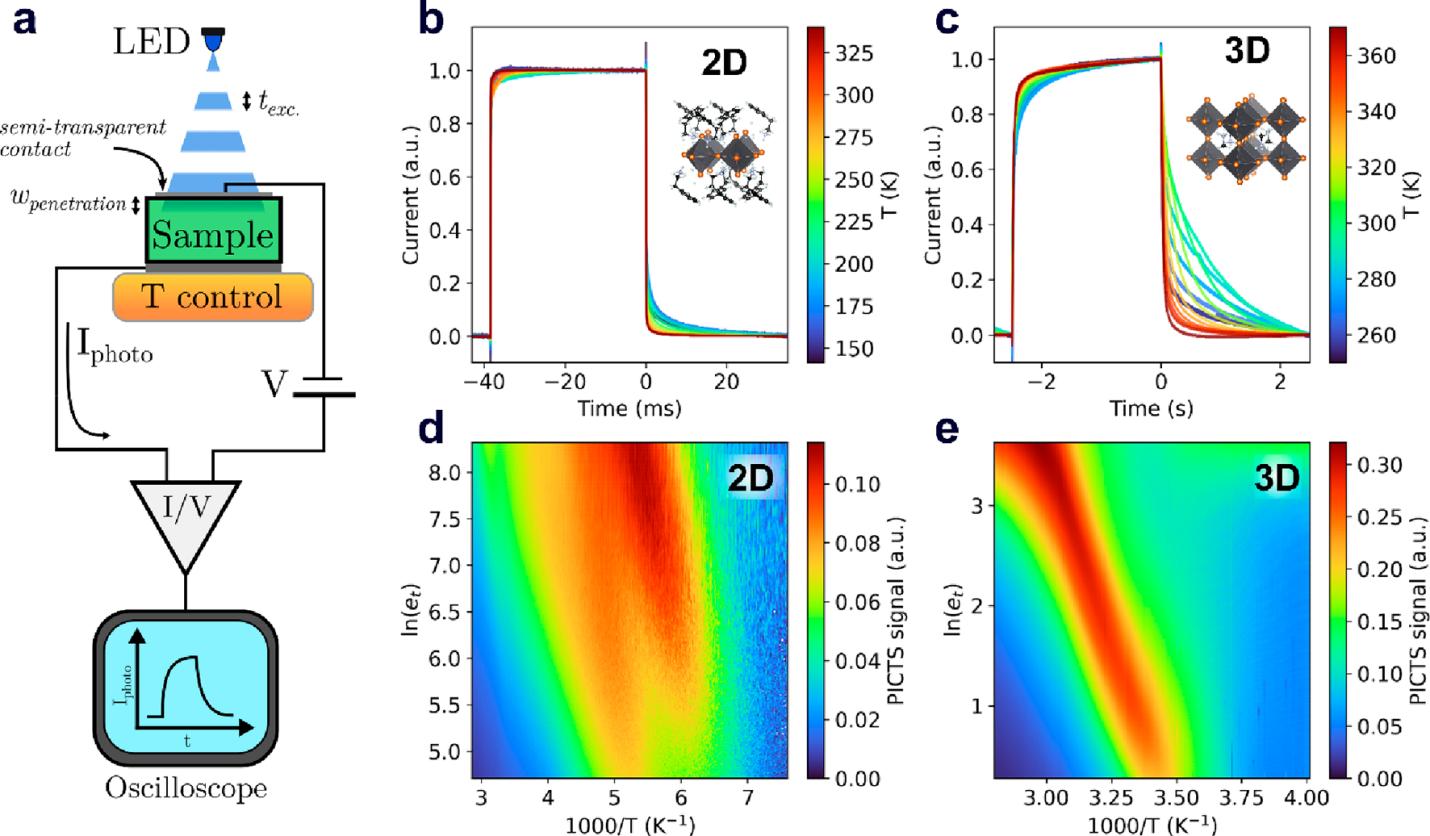
Photoinduced current transient spectroscopy (PICTS) on 2D-like and standard metal halide perovskite. (a) PICTS experimental setup: a power supply is connected to an MHP single crystal to impose an external bias voltage across the sample; an intermittent (box-shaped), above bandgap light impinging on the MHP is absorbed in a depth *w*_penetration_ generating photocurrent transients; the current dynamic is recorded via a current amplifier whose output is read by a digital oscilloscope. (b and c) (PEA)_2_PbBr_4_ (b) and MAPbBr_3_ (c) normalized current transients over a given temperature range highlighted with a color map. The (PEA)_2_PbBr_4_ transients are about 3 orders of magnitude faster. (d and e) (PEA)_2_PbBr_4_ (d) and MAPbBr_3_ (e) PICTS signals plotted versus inverse temperature, corresponding to the transients shown in panels b and c, respectively. High PICTS signals (red color) correspond to higher recorded currents at a given temperature in the time interval considered (see the Supporting Information for details).

In any case, if the ions have a major impact on the photocurrent modulation, we argue that the photocurrent transients will be ruled not by the elemental charge carriers’ detrapping dynamics (relatively fast) but by the ion dynamics (relatively slow). In this case the interpretation of the PICTS signals shifts from a description of electronic trap states (like in the 2D MHPs, Figure 1d) to a description of ion drift parameters (as will be shown for the 3D MHPs data reported in Figure 1e). In the following, we focus on the latter, while leaving an in-depth discussion of the PICTS analysis of the (PEA)_2_PbBr_4_ trap states to sister paper jointly submitted.^21^

To support our claim, we report impedance spectroscopy (IS) data strongly suggesting photoenhanced ion migration. Then, we discuss the frequency-dependent contributions to the photocurrents in the 2D and the 3D MHPs, obtained by intensity modulated photocurrent spectroscopy (IMPS) (Figure S3). Figure 2a,b shows IS spectra for the 3D and 2D MHPs considered, highlighting the variation of impedance between the dark and the above bandgap illumination conditions for the two materials. As the light is turned on, the impedance in the two semiconductors drops due to the excess of photogenerated charge carriers. However, in contrast to the 2D MHP, the 3D MHP low-frequency impedance consistently evolves over time (Figure 2a,b inset), suggesting a photoinduced ion migration. By fitting the dynamics with exponential decays, we find a time constant of ∼3000 s. This value corresponds to an average ion diffusivity of ∼2 × 10^–8^ cm^2^/s (due to light-induced ion drift throughout the entire crystal thickness), falling in the literature reported ranges of 10^–5^ and 10^–9^ cm^2^ V^–1^ s^–1^ for ionic species (see Figure S3 and the Supporting Information discussion for calculations).^7^

**Figure 2:**
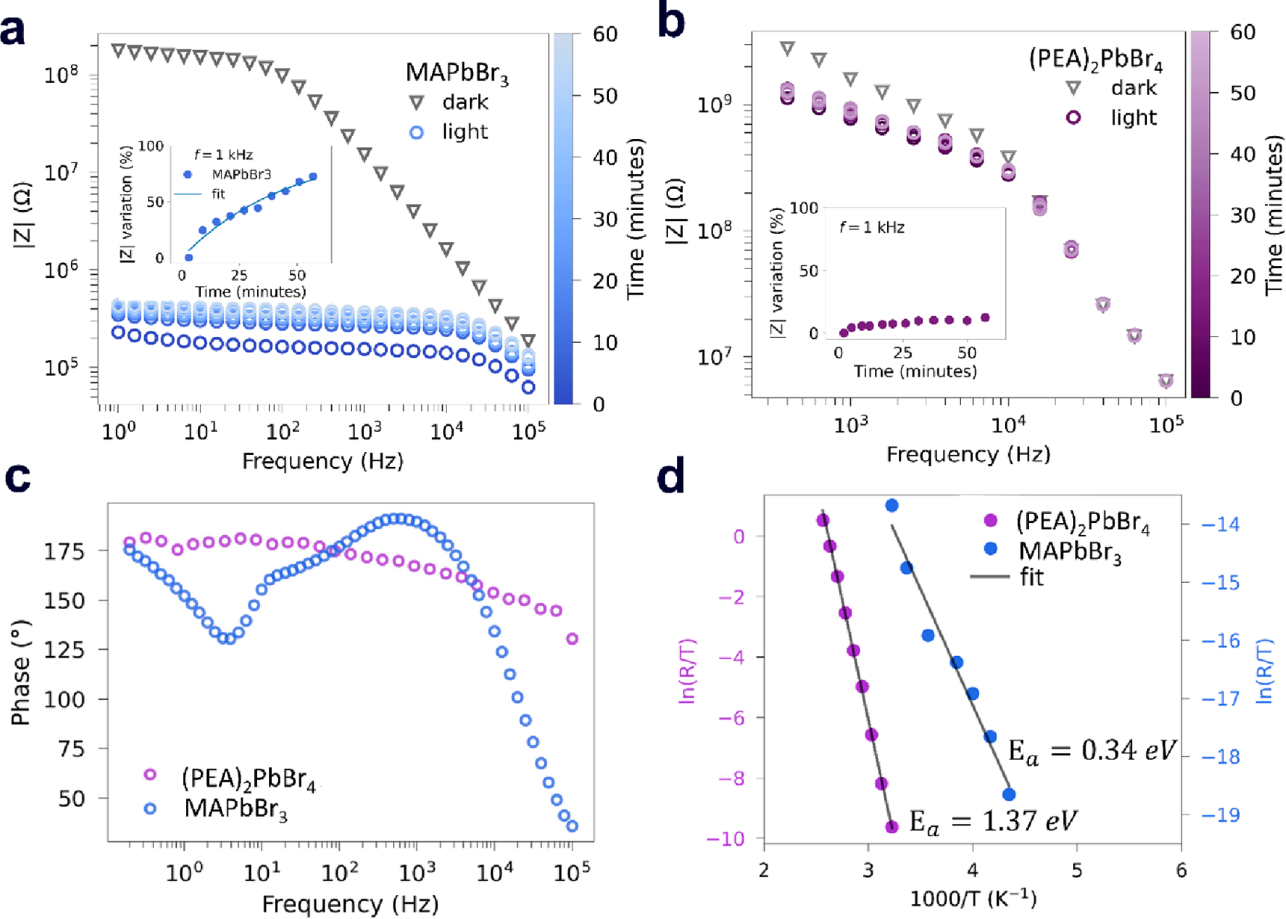
(a and b) Impedance spectroscopy (IS) measurements at 5 V bias, under dark and above bandgap illumination (5 mW/cm^2^) conditions for the MAPbBr_3_ (a) and (PEA)_2_PbBr_4_ (b). In the insets, the respective impedance modules over time for fixed frequencies are plotted. (c and d) Intensity modulated photocurrent spectroscopy (IMPS) (c) and dark current versus temperature (d) on MAPbBr_3_ (blue dots) and (PEA)_2_PbBr_4_ (purple dots). The IMPS photocurrent dephasing at a low frequency is associated with a slow ionic response. Dark conductivity versus temperature (d) in MAPbBr_3_ and (PEA)_2_PbBr_4_ elaborated from data reported in Figure S5.

Confirmation that ions play a leading role in the photocurrent modulation at low frequencies in 3D MHPs can be inferred from IMPS results showing a light-induced perturbation change in the photocurrent generation (Figure 2c). The analysis performed on the IMPS data (see the Supporting Information discussion for details) highlights a clear (and dominant, see Nyquist plot Figure S4) photocurrent contribution in the 0.1–500 Hz range for the MAPbBr_3_ that is absent in the (PEA)_2_PbBr_4_.

Finally, current versus voltage measurements over temperature were performed on both samples (Figure S5), and the derived conductivity versus temperature plot (Figure 2d) was fit to extract the ionic conductivity activation energy.^22^ The absolute difference between these values further confirms a large ionic impact discrepancy in these two materials.

To uncover the physical mechanisms underlying the photocurrent generation, we consider the frequency range where the most photocurrent generation occurs, and we assume photochemical ionization as the only plausible *direct* ionic contribution to the photocurrent. In this process, the photogenerated charge carriers make the ions metastable. The ions then induce a strain in the perovskite structure, and the carrier generation gradient causes light-induced ion migration.^23−25^ For instance, the photogenerated holes can be trapped by a Br becoming interstitial, yielding to the following reaction, expressed in Kröger–Vink notation:^24,26^*Br*_*Br*_^*x*^ + *h*^•^ ⇄ *Br*_*i*_^*x*^ + *V*_*Br*_^•^. Nevertheless, considering the several orders of magnitude difference in carrier mobility for ions and electrons (or holes), and their commensurate concentration under illumination,^20^ we tend to exclude the photochemical ionization as major responsible for the photocurrent modulation. This consideration particularly applies to single crystals, in the absence of grain boundary effects where ion migration activation energy is relatively higher.^10,27^

Therefore, the results reported in Figure 2 strongly suggest that in 3D MHPs, in contrast to 2D MHPs, ions have an *indirect*, but major, impact on the photogenerated charge carriers in the 0.1–100 Hz frequency range. Since the photocurrent transient decay times are approximately 1–10 s long, we expect them to be governed by ion movement. This would also reasonably explain the peculiar slow dynamics observed in both the photocurrent decay and rise (cf. Figure 1e) that does not fit in the classic PICTS interpretation.^11,12^ Furthermore, it has been observed that ion migration, albeit much slower than the electronic one,^18^ can modify the local extent of electrical doping, thus modulating the total current density.^28,29^ Nevertheless, while a direct proportionality between light intensity, ionic diffusion coefficient,^30^ and mobile ion concentration^8^ has been already reported, a model to describe the photocurrent transient caused by intermittent illumination at fixed bias voltage is still missing.^31^

Therefore, we developed a physical model to interpret the ion drift-governed PICTS photocurrent transients in MAPbBr_3_. Figure 3a shows the band structure diagram of the metal–perovskite heterojunction. At zero bias, the MHPs valence and conduction bands bend downward due to the presence of fixed positive charges at the metal–perovskite interfaces (left and right contacts). This effect is small enough not to be observed in a current–voltage sweep (showing rather Ohmic behavior, see Figure S5c,d), but it was appreciated with spatially resolved photocurrent spectroscopies.^32,33^ In dark conditions, mobile charges^18^ screen the interfacial (fixed) charges, creating a negative space charge region (SCR) *W*_*D*_ extending for several microns back in the bulk.^33^ Upon application of negative voltage (Figure 3b), holes and positive mobile ions move toward the semitransparent (left) contact; while holes are extracted from the electrodes and recorded as dark current, ions accumulate at the perovskite–metal interface, thus modifying the band bending. In fact, mobile positive ions bring further positive charge to the negative contact, enlarging the SCR. When an above bandgap light pulse illuminates the semitransparent (left) metal contact (Figure 3c), electron–hole pairs are generated in a few hundred nanometers inside the MHPs. Due to the downward band bending, the photogenerated electrons remain localized at the interface and get trapped by the fixed interfacial positive ions. The localized electrons are screened by holes attracted from the external circuit to the left contact−perovskite interface. This hole current in the external circuit causes the reverse current spike observed in the microsecond time scale (see Figure S6).^34,35^ After the spike, photogenerated electrons neutralize the fixed charges flattening the bands, as also observed in photomapping experiments.^33^ The positive mobile ions accumulated at the left contact move in response to such a light-induced change in band bending. Since the slope of the band changes from positive to negative, they are accelerated toward the left contact. When the light is turned off, the trapped electrons at the interface recombine with holes in the valence bands. Thus, an additional current of holes flows to the external circuit in the opposite direction, yielding a reverse spike of opposite sign (Figure S6). As a result, the fixed positive charges at the interface are no longer neutralized, and the bands go back to the downward bending condition. In response to this local change in the band, the mobile positive ions are pulled back toward the bulk. When the bias is reversed, i.e., a positive bias is applied to the semitransparent left electrode through which the sample illumination occurs, the situation is not symmetric (Figure S7). Due to the bias, negatively charged mobile ions accumulate at the left contact, but upon photoexcitation, the trapped carriers at the interface cause smaller band banding variation, as the bands’ slope does not change sign. Under positive polarization indeed, reverse current spikes are not observed (Figure S7). The mobile negative ions drift toward the bulk under illumination and then return to the original position after the dark condition is restored.

**Figure 3:**
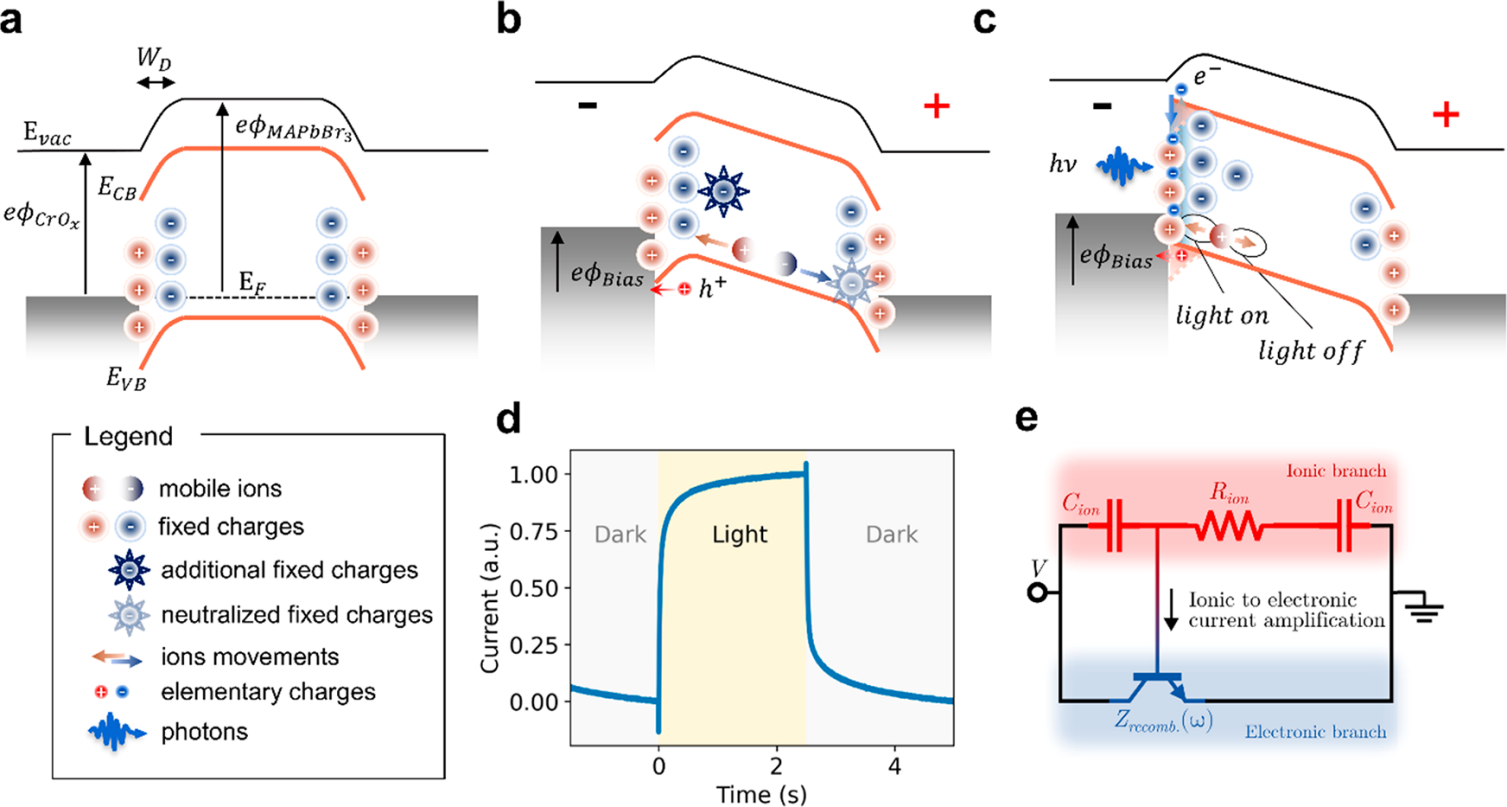
(a–c) Photocurrent model to describe transient photocurrent in MAPbBr_3_ where ions dominate the charge transport. Band diagram of 3D MHP with metal contacts where energy levels are represented on the vertical axis and the device out-of-plane direction corresponds to the horizontal axis (see main text for description). (d) Experimental photocurrent transient at room temperature. A 470 nm LED (fluence of 5 mW/cm^2^) is switched ON at *t* = 0 and switched OFF at *t* = 2.5 s. (e) Equivalent circuit of the metal–MHP interface showing two separate conductivity branches, ionic and electronic, connected via the bipolar junction transistor (BJT) transistor gate regulated by the ionic part of the circuit.^18^

In short, trap state filling by photogenerated charges causes change in the band bending, and ions migrate in response to such change. To analyze the impact of the ion redistribution on the electron and hole photocurrent transients we refer to the equivalent circuit model represented in Figure 3e.^18^ The upper branch of the circuit represents the ionic contribution, which is characterized by two capacitors at the two contact interfaces *C*_ion_ and a bulk ionic resistance *R*_ion_. The lower branch represents the electronic contribution, which is modeled by the electronic recombination impedance *Z*_rec_ (typically a recombination resistance and capacitance in parallel). As described in ref (18), *Z*_rec_ can be modeled as a bipolar transistor whose gate is controlled by the ionic circuit branch. This means that ion movement can amplify the current that flows in the electronic circuit branch. Note that *Z*_rec_ is frequency-dependent, and the frequency can be modulated by slow-moving ionic species.

Our model can explain the unexpectedly slow photocurrent transients in 3D MHPs arising from a highly stable interaction of the photogenerated charged carriers with the ions.^36^ (Figures 1b, 4, and S8). Therefore, by analyzing the transients we expect to extract ion drift parameters by exploiting the light-induced modulation of the metal–perovskite built-in electrical field, even though the photocurrents are generated by the elementary charge carriers and not by ions (see the Supporting Information discussion).^19,37,38^

**Figure 4:**
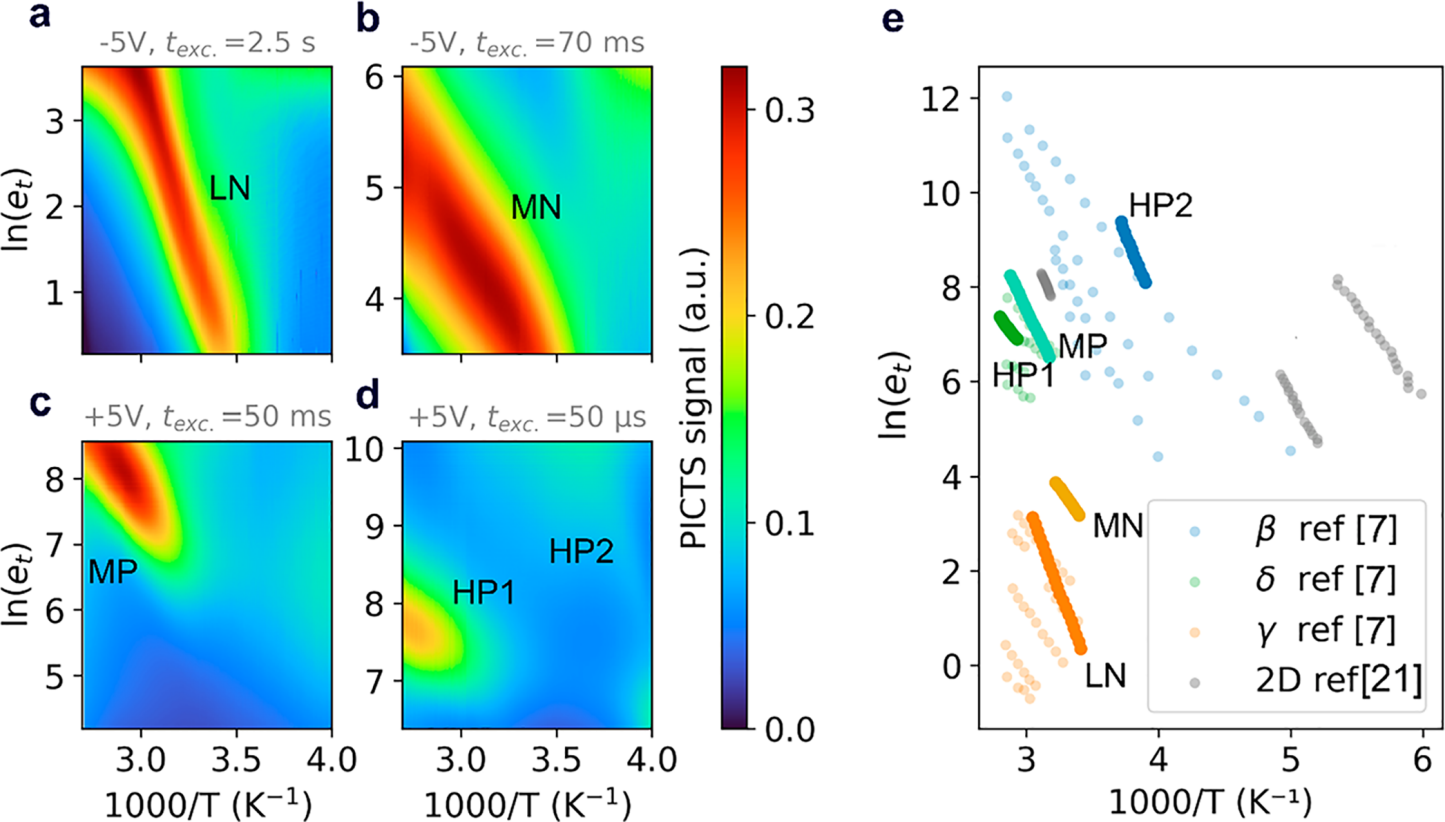
(a–d) PICTS maps referred to the MAPbBr_3_ single crystal contacted with a top-bottom configuration and illuminated through the semitransparent top contact. The maps are obtained for negative (a and b) and positive (c and d) voltages with different light pulse excitation times: 2.5 s (a); 70 ms (b); 50 ms (c); 50 μs (d). (e) PICTS signal maxima (shown as red color in maps a–d) plotted against inverse temperature (Arrhenius plots) to highlight the slopes proportional to the ion activation energies and comparison with ionic defects reported in refs (7) and (21).

With this model in hand, we interpret the results of the PICTS measurements in MAPbBr_3_ single crystals. The use of positive (Figure 4a,b) and negative (Figure 4c,d) biases at the illuminated semitransparent contact allows us to investigate separately the negative and positive ion migration, respectively (see the Supporting Information discussion). Moreover, we introduced frequency-dependent PICTS experiments where we varied the photoexcitation times from seconds to microseconds to characterize ionic species with different diffusivity value ranges and eventually retrieve electronic characterization at frequencies where the ionic contribution becomes negligible. The results of these experiments are reported in Figure 4e where PICTS maxima at low, medium, and high frequencies (L, M, H) with positive (P) or negative (N) voltages are accordingly labeled and in case of multiple peaks within the same experiment, numbered (e.g., LN is the only peak retrieved at low frequency, applying a negative bias).

Then we looked for previously reported Arrhenius plots regarding ion diffusion in MHPs, and we compared them to our results (Figure 4e) by using the Einstein relationship linking drifting and diffusion (see the Supporting Information discussion). Noting a remarkable agreement with the values and ion charge signs reported by Deibel’s group on MAPbI_3_ TID,^7^ we carry on a tentative ionic species assignment following their approach (see the Supporting Information for calculations related to Table 1 values).

**Table 1:** Experimental Values (from Figure 4e) of Ion Activation Energies *E*_a_ Extracted by PICTS Compared with Literature Results^7^ and Calculated Diffusion Coefficient *D*_300K_ (see the Supporting Information)^a^

Peak label	*E*_a_ (eV)	*D*_300K_ (cm^2^/s)	Tentative assignment
LN	0.67 ± 0.03	≈10^–9^	MA_*i*_^•^
MN	0.37 ± 0.018	≈10^–8^	*V*_*Br*_^•^
MP	0.53 ± 0.03	≈10^–7^	Br_*i*_^′^
HP1	0.34 ± 0.017	≈10^–7^	Br_*i*_^′^
HP2	0.62 ± 0.03	≈10^–4^	*V*_*MA*_^′^/electronic

a The peaks are labeled with two letters according to the light pulse frequency (low – L, medium – M, high – H), and the sign of the applied voltage (N – negative, P – positive); a number is used to distinguish multiple peaks within the same PICTS map.

The activation energies for ion migration, comparable to ion conductors,^39,40^ reported in Table 1 fall within the range reported by several groups on a wide range of MHPs (∼0.2–0.7 eV),^6,7,13,41−43^ and this agreement extends to the estimated diffusion coefficient.^7,38,44,45^ In particular, LN, MN, MP, and HP1 fall in the range of 10^–7^–10^–9^ cm^2^s^–1^ reported on MAPbBr_3_ single crystals.^19,20^ Moreover the lowest activation energy retrieved from PICTS (0.34 eV) corresponds to the activation energy calculated from current–voltage plots (Figure 2d). The only result diverging from the literature is HP2, which in our model yields a diffusion coefficient of approximately 10^–4^ cm^2^ s^–1^. Such high diffusivity could be a symptom of a different charge emission mechanism, such as electron trapping, which could be possibly appreciated by the high-frequency measurements. Further investigation is needed to assess the origin of the HP2 trace.

Ion migration in MHPs under illumination is a complex phenomenon, and an in-depth understanding of it under operating conditions is of fundamental importance, since most applications of perovskite-based devices imply exposure to light during operation (LEDs, solar cells, and photodetectors). Here, ions and their relative vacancies may share the same activation energies^2^ and they can become more mobile due to a variation in the local chemical potential, i.e., when the free charge carrier concentrations changes upon light absorption.^8,23,30,46^ To study charge transport in this context, we adapted the PICTS, a photocurrent defect spectroscopy technique previously used on inorganic semiconductors, to 2D and 3D MHPs, reporting the detection of electronic defects states in (PEA)_2_PbBr_4_.^21^ On MAPbBr_3_ instead, where ions are highly mobile, we demonstrated that the extracted PICTS parameters are to be interpreted in terms of ion diffusivity values. Our observations help distinguish different ion migration activation energies and diffusion coefficient linked to a light stimulus instead of a voltage stimulus as it occurs in TID. Despite this intrinsic difference, we showed that the two techniques unexpectedly yield similar results and that their connection is mediated by the photoinduced modulation of the built-in metal–MHP field. Our results put forward an alternative technique for the extraction of ion diffusion parameters and at the same time pose limitations to the interpretation of PICTS signals related to trap states, in materials where ion migration governs the photocurrent extraction.

## Experimental Section

### Synthesis of MAPbBr_3_

The MAPbBr_3_ was synthesized according to previously reported procedure.^47^ Briefly, a 1 M growth solution was prepared by dissolving stoichiometric amounts of MABr (Great Cell Solar, ≥99%) and PbBr_2_ (Sigma-Aldrich, anhydrous 99.999%) precursors in N,N-Dimethylformamide (DMF, Sigma-Aldrich, anhydrous, 99.8%). Crystals were grown from seeds of about 1 mm in width, with a perfect shape. Crystallization was triggered and controlled by applying a temperature ramp to 3.5 mL of the growth solution containing the seed, from 62.6 °C up to 81.5 °C with an average temperature increase of 3.2 °C/h.

Metal contacts were deposited across the crystal by thermal evaporation of chromium on the top and bottom sizes of the {001} crystal planes (30 nm CrO_*x*_ top semitransparent contact, 80 nm CrO_*x*_ bottom contact). During evaporation the slightly oxidizing atmosphere (*p* ≈ 10^–5^ mbar) promoted the formation of the low-reacting, ion-blocking, and nondiffusive CrO_*x*_ layer that furthermore provided a good hole-injection efficiency into the MHP valence band.^48^

### Synthesis of (PEA)_2_PbBr_4_

The (PEA)_2_PbBr_4_ was synthesized according to previously reported procedure.^49^ Briefly, the as-purchased precursors PEABr and PbBr_2_ (1:1 molar ratio) were dissolved in 5 mL of DMF to obtain a 1.3 M solution. After overnight stirring, the solution was pushed through a 0.22 μm PFTE filter and allowed to rest in a beaker partially covered with parafilm. Over the course of 2 weeks, as the DMF slowly evaporated, a (PEA)_2_PbBr_4_ single crystal formed inside the solution. The crystal was extracted from the beaker and blow-dried.

The (PEA)_2_PbBr_4_ crystals were contacted by CrO_*x*_ thermal evaporation through a shadow mask, achieving a channel length of 100 μm and width of 1 mm, in a coplanar configuration with respect to the crystal (001) surface. This configuration allowed achieving a measurable current that was otherwise not detectable in the stacked contact configuration.

### Impedance Spectroscopy

To perform impedance spectroscopy (IS) we used the same samples employed for PICTS and mounted them in a Nextron vacuum chamber equipped with optical access to the sample and electrical contacts. Blue light (470 nm) and UV light (365 nm) was used to excite over bandgap the MAPbBr_3_ and (PEA)_2_PbBr_4_ crystals, respectively. The samples were kept under illumination and at 0 V applied bias for the entire duration of the experiment (1 h). The evolution of the impedance of the sample over time was monitored every 6 min using an MFLI lock-in amplifier (from Zurich Instruments). A constant DC offset voltage of 5 V and sinusoidal oscillation were applied between the top and bottom electrical contacts of the sample. The amplitude of oscillation was set to 200 mV, and the frequencies were in the 1 Hz–100 kHz range for MAPbBr_3_ and in the 100 Hz–100 kHz range for (PEA)_2_PbBr_4_. The spectrum below 100 Hz on (PEA)_2_PbBr_4_ becomes too noisy due to the very high impedance of the sample; therefore, it is not shown here.

### Intensity Modulated Photocurrent Spectroscopy

To perform intensity modulated photocurrent spectroscopy (IMPS) we used the same samples employed for PICTS and we mounted them in the same Nextron vacuum chamber and kept at a constant applied bias of 7 and 20 V for the MAPbBr_3_ and (PEA)_2_PbBr_4_ crystals, respectively. The voltages were set at a useful minimum to extract a good signal-to-noise current ratio. Blue light (470 nm) and UV light (365 nm) with a DC intensity of 5 and 1.3 mW/cm^2^ were used to excite over bandgap the MAPbBr_3_ and (PEA)_2_PbBr_4_ crystals, respectively. The two LEDs were driven by an LED driver (Thorlabs DC2100) to superimpose a sinusoidal modulated illumination of about 10% of the illumination bias, to ensure a linear response of the LEDs, in a frequency range of 0.1 Hz–100 kHz. Using a beam splitter, the light was divided into two beams, one on the chamber to illuminate the sample and the other one on a calibrated Si photodiode (Hamamatsu) to measure the light intensity. The two signals (the photocurrent and the light intensity on the photodiode) were collected simultaneously by the Frequency Response Analyzer (FRA32M) module of a PGSTAT204 electrochemical workstation.

### Photoinduced Current Transient Spectroscopy

To perform photoinduced current transient spectroscopy (PICTS) measurements, the samples equipped with metallic contacts were connected by use of silver paste and gold microwires to thermally decoupled copper leads and then to the external circuit. The circuit was used to supply the voltage and record current transients via a current amplifier. The sample temperature was controlled via a cryogenic system, and external pulsed light sources were used to generate photocurrents in the samples. The photocurrent modulation was obtained by an above bandgap sample illumination driven with a square current signal. For an effective data visualization we readapt a recently proposed technique for PICTS data analysis^50^ used also for representing transient ion drift (TID) results on MHPs.^7^ The stacked contact configuration allowed us to investigate separately both negative and positive charge effects in a MAPbBr_3_ during PICTS experiments, in opposition to the coplanar configuration adopted on the (PEA)_2_PbBr_4_. The sample was wired and loaded in a cryogenic chamber for performing the IMPS and PICTS measurements in a rough vacuum (∼10^–3^ mbar).

## Supporting Information Available

Samples’ photographs and materials’ atomic structure; impedance spectroscopy (IS) setup and data; intensity modulated photocurrent spectroscopy (IMPS) setup and data; *I*–*V* curves over temperature; photocurrent transients’ data and charge drift modeling at the semitransparent electrode; repeatability and stability measurements; coplanar contacts photocurrent transients; ion mobility calculations; PICTS simulation and discussion; ion assignment discussion (PDF)

## References

[ref1] Wei H.; Huang J. Halide Lead Perovskites for Ionizing Radiation Detection. Nat. Commun. 2019, 10 (1), 1066 10.1038/s41467-019-08981-w. 30842411 PMC6403296

[ref2] Duan L.; Uddin A. Defects and Stability of Perovskite Solar Cells: A Critical Analysis. Mater. Chem. Front. 2022, 6 (4), 400–417. 10.1039/D1QM01250A.

[ref3] Meggiolaro D.; De Angelis F. First-Principles Modeling of Defects in Lead Halide Perovskites: Best Practices and Open Issues. ACS Energy Lett. 2018, 3 (9), 2206–2222. 10.1021/acsenergylett.8b01212.

[ref4] Lang D. V. Deep-level Transient Spectroscopy: A New Method to Characterize Traps in Semiconductors. J. Appl. Phys. 1974, 45 (7), 3023–3032. 10.1063/1.1663719.

[ref5] Heo S.; Seo G.; Lee Y.; Lee D.; Seol M.; Lee J.; Park J.-B.; Kim K.; Yun D.-J.; Kim Y. S.; Shin J. K.; Ahn T. K.; Nazeeruddin M. K. Deep Level Trapped Defect Analysis in CH_3_NH_3_PbI_3_ Perovskite Solar Cells by Deep Level Transient Spectroscopy. Energy Environ. Sci. 2017, 10 (5), 1128–1133. 10.1039/C7EE00303J.

[ref6] Futscher M. H.; Lee J. M.; McGovern L.; Muscarella L. A.; Wang T.; Haider M. I.; Fakharuddin A.; Schmidt-Mende L.; Ehrler B. Quantification of Ion Migration in CH_3_NH_3_PbI_3_ Perovskite Solar Cells by Transient Capacitance Measurements. Mater. Horiz. 2019, 6 (7), 1497–1503. 10.1039/C9MH00445A.

[ref7] Reichert S.; An Q.; Woo Y. W.; Walsh A.; Vaynzof Y.; Deibel C. Probing the Ionic Defect Landscape in Halide Perovskite Solar Cells. Nat. Commun. 2020, 11 (1), 6098 10.1038/s41467-020-19769-8. 33257707 PMC7705665

[ref8] McGovern L.; Grimaldi G.; Futscher M. H.; Hutter E. M.; Muscarella L. A.; Schmidt M. C.; Ehrler B. Reduced Barrier for Ion Migration in Mixed-Halide Perovskites. ACS Appl. Energy Mater. 2021, 4 (12), 13431–13437. 10.1021/acsaem.1c03095. 34977472 PMC8715422

[ref9] Reichert S.; Flemming J.; An Q.; Vaynzof Y.; Pietschmann J.-F.; Deibel C. Ionic-Defect Distribution Revealed by Improved Evaluation of Deep-Level Transient Spectroscopy on Perovskite Solar Cells. Phys. Rev. Appl. 2020, 13 (3), 034018 10.1103/PhysRevApplied.13.034018.

[ref10] Tammireddy S.; Reichert S.; An Q.; Taylor A. D.; Ji R.; Paulus F.; Vaynzof Y.; Deibel C. Temperature-Dependent Ionic Conductivity and Properties of Iodine-Related Defects in Metal Halide Perovskites. ACS Energy Lett. 2022, 7 (1), 310–319. 10.1021/acsenergylett.1c02179.

[ref11] Balland J. C.; Zielinger J. P.; Noguet C.; Tapiero M. Investigation of Deep Levels in High-Resistivity Bulk Materials by Photo-Induced Current Transient Spectroscopy. I. Review and Analysis of Some Basic Problems. J. Phys. Appl. Phys. 1986, 19 (1), 57–70. 10.1088/0022-3727/19/1/011.

[ref12] Balland J. C.; Zielinger J. P.; Tapiero M.; Gross J. G.; Noguet C. Investigation of Deep Levels in High-Resistivity Bulk Materials by Photo-Induced Current Transient Spectroscopy. II. Evaluation of Various Signal Processing Methods. J. Phys. Appl. Phys. 1986, 19 (1), 71–87. 10.1088/0022-3727/19/1/012.

[ref13] Pecunia V.; Zhao J.; Kim C.; Tuttle B. R.; Mei J.; Li F.; Peng Y.; Huq T. N.; Hoye R. L. Z.; Kelly N. D.; Dutton S. E.; Xia K.; MacManus-Driscoll J. L.; Sirringhaus H. Assessing the Impact of Defects on Lead-Free Perovskite-Inspired Photovoltaics via Photoinduced Current Transient Spectroscopy. Adv. Energy Mater. 2021, 11 (22), 2003968 10.1002/aenm.202003968.

[ref14] Futscher M. H.; Deibel C. Defect Spectroscopy in Halide Perovskites Is Dominated by Ionic Rather than Electronic Defects. ACS Energy Lett. 2022, 7 (1), 140–144. 10.1021/acsenergylett.1c02076.

[ref15] Senocrate A.; Maier J. Solid-State Ionics of Hybrid Halide Perovskites. J. Am. Chem. Soc. 2019, 141 (21), 8382–8396. 10.1021/jacs.8b13594. 31017426 PMC6727625

[ref16] Guerrero A.; Bisquert J.; Garcia-Belmonte G. Impedance Spectroscopy of Metal Halide Perovskite Solar Cells from the Perspective of Equivalent Circuits. Chem. Rev. 2021, 121 (23), 14430–14484. 10.1021/acs.chemrev.1c00214. 34845904

[ref17] Pockett A.; Carnie M. J. Ionic Influences on Recombination in Perovskite Solar Cells. ACS Energy Lett. 2017, 2 (7), 1683–1689. 10.1021/acsenergylett.7b00490.

[ref18] Moia D.; Gelmetti I.; Calado P.; Fisher W.; Stringer M.; Game O.; Hu Y.; Docampo P.; Lidzey D.; Palomares E.; Nelson J.; Barnes P. R. F. Ionic-to-Electronic Current Amplification in Hybrid Perovskite Solar Cells: Ionically Gated Transistor-Interface Circuit Model Explains Hysteresis and Impedance of Mixed Conducting Devices. Energy Environ. Sci. 2019, 12 (4), 1296–1308. 10.1039/C8EE02362J.

[ref19] García-Batlle M.; Baussens O.; Amari S.; Zaccaro J.; Gros-Daillon E.; Verilhac J.; Guerrero A.; Garcia-Belmonte G. Moving Ions Vary Electronic Conductivity in Lead Bromide Perovskite Single Crystals through Dynamic Doping. Adv. Electron. Mater. 2020, 6 (10), 2000485 10.1002/aelm.202000485.

[ref20] García-Batlle M.; Mayén Guillén J.; Chapran M.; Baussens O.; Zaccaro J.; Verilhac J.-M.; Gros-Daillon E.; Guerrero A.; Almora O.; Garcia-Belmonte G. Coupling between Ion Drift and Kinetics of Electronic Current Transients in MAPbBr_3_ Single Crystals. ACS Energy Lett. 2022, 7 (3), 946–951. 10.1021/acsenergylett.1c02578. 35310458 PMC8922277

[ref21] Ciavatti A.; Foderà V.; Armaroli G.; Maserati L.; Fraboni B.; Cavalcoli D. Radiation Hardness and Defects Activity in PEA_2_PbBr_4_ Single Crystals. arXiv, 10.48550/arXiv.2309.13355.

[ref22] Lee H.; Gaiaschi S.; Chapon P.; Tondelier D.; Bourée J.-E.; Bonnassieux Y.; Derycke V.; Geffroy B. Effect of Halide Ion Migration on the Electrical Properties of Methylammonium Lead Tri-Iodide Perovskite Solar Cells. J. Phys. Chem. C 2019, 123 (29), 17728–17734. 10.1021/acs.jpcc.9b04662.

[ref23] Motti S. G.; Meggiolaro D.; Barker A. J.; Mosconi E.; Perini C. A. R.; Ball J. M.; Gandini M.; Kim M.; De Angelis F.; Petrozza A. Controlling Competing Photochemical Reactions Stabilizes Perovskite Solar Cells. Nat. Photonics 2019, 13 (8), 532–539. 10.1038/s41566-019-0435-1.

[ref24] Kim G. Y.; Senocrate A.; Yang T.-Y.; Gregori G.; Grätzel M.; Maier J. Large Tunable Photoeffect on Ion Conduction in Halide Perovskites and Implications for Photodecomposition. Nat. Mater. 2018, 17 (5), 445–449. 10.1038/s41563-018-0038-0. 29555997

[ref25] Tirmzi A. M.; Christians J. A.; Dwyer R. P.; Moore D. T.; Marohn J. A. Substrate-Dependent Photoconductivity Dynamics in a High-Efficiency Hybrid Perovskite Alloy. J. Phys. Chem. C 2019, 123 (6), 3402–3415. 10.1021/acs.jpcc.8b11783.

[ref26] De Souza R.; Harrington G. Revisiting Point Defects in Ionic Solids and Semiconductors. Nat. Mater. 2023, 22 (7), 794–797. 10.1038/s41563-023-01583-4. 37386062

[ref27] McGovern L.; Koschany I.; Grimaldi G.; Muscarella L. A.; Ehrler B. Grain Size Influences Activation Energy and Migration Pathways in MAPbBr3 Perovskite Solar Cells. J. Phys. Chem. Lett. 2021, 12 (9), 2423–2428. 10.1021/acs.jpclett.1c00205. 33661008 PMC8041307

[ref28] Jacobs D. A.; Shen H.; Pfeffer F.; Peng J.; White T. P.; Beck F. J.; Catchpole K. R. The Two Faces of Capacitance: New Interpretations for Electrical Impedance Measurements of Perovskite Solar Cells and Their Relation to Hysteresis. J. Appl. Phys. 2018, 124 (22), 225702 10.1063/1.5063259.

[ref29] Li C.; Guerrero A.; Huettner S.; Bisquert J. Unravelling the Role of Vacancies in Lead Halide Perovskite through Electrical Switching of Photoluminescence. Nat. Commun. 2018, 9 (1), 5113 10.1038/s41467-018-07571-6. 30504825 PMC6269531

[ref30] Xing J.; Wang Q.; Dong Q.; Yuan Y.; Fang Y.; Huang J. Ultrafast Ion Migration in Hybrid Perovskite Polycrystalline Thin Films under Light and Suppression in Single Crystals. Phys. Chem. Chem. Phys. 2016, 18, 30484 10.1039/C6CP06496E. 27782266

[ref31] Bou A.; Pockett A.; Cruanyes H.; Raptis D.; Watson T.; Carnie M. J.; Bisquert J. Limited Information of Impedance Spectroscopy about Electronic Diffusion Transport: The Case of Perovskite Solar Cells. APL Mater. 2022, 10 (5), 051104 10.1063/5.0087705.

[ref32] Ahmadi M.; Collins L.; Higgins K.; Kim D.; Lukosi E.; Kalinin S. V. Spatially Resolved Carrier Dynamics at MAPbBr_3_ Single Crystal–Electrode Interface. ACS Appl. Mater. Interfaces 2019, 11 (44), 41551–41560. 10.1021/acsami.9b16287. 31595742

[ref33] Shrestha S.; Tsai H.; Yoho M.; Ghosh D.; Liu F.; Lei Y.; Tisdale J.; Baldwin J.; Xu S.; Neukirch A. J.; Tretiak S.; Vo D.; Nie W. Role of the Metal–Semiconductor Interface in Halide Perovskite Devices for Radiation Photon Counting. ACS Appl. Mater. Interfaces 2020, 12 (40), 45533–45540. 10.1021/acsami.0c11805. 32886475

[ref34] Ebadi F.; Taghavinia N.; Mohammadpour R.; Hagfeldt A.; Tress W. Origin of Apparent Light-Enhanced and Negative Capacitance in Perovskite Solar Cells. Nat. Commun. 2019, 10 (1), 1574 10.1038/s41467-019-09079-z. 30952882 PMC6450882

[ref35] Hernández-Balaguera E.; Bisquert J. Negative Transient Spikes in Halide Perovskites. ACS Energy Lett. 2022, 7 (8), 2602–2610. 10.1021/acsenergylett.2c01252.

[ref36] Motti S. G.; Meggiolaro D.; Martani S.; Sorrentino R.; Barker A. J.; De Angelis F.; Petrozza A. Defect Activity in Lead Halide Perovskites. Adv. Mater. 2019, 31 (47), 1901183 10.1002/adma.201901183. 31423684

[ref37] Pockett A.; Eperon G. E.; Sakai N.; Snaith H. J.; Peter L. M.; Cameron P. J. Microseconds, Milliseconds and Seconds: Deconvoluting the Dynamic Behaviour of Planar Perovskite Solar Cells. Phys. Chem. Chem. Phys. 2017, 19 (8), 5959–5970. 10.1039/C6CP08424A. 28177002

[ref38] Wang H.; Guerrero A.; Bou A.; Al-Mayouf A. M.; Bisquert J. Kinetic and Material Properties of Interfaces Governing Slow Response and Long Timescale Phenomena in Perovskite Solar Cells. Energy Environ. Sci. 2019, 12 (7), 2054–2079. 10.1039/C9EE00802K.

[ref39] Eames C.; Frost J. M.; Barnes P. R. F.; O’Regan B. C.; Walsh A.; Islam M. S. Ionic Transport in Hybrid Lead Iodide Perovskite Solar Cells. Nat. Commun. 2015, 6 (May), 2–9. 10.1038/ncomms8497. PMC4491179 26105623

[ref40] Kosasih F. U.; Ducati C. Characterising Degradation of Perovskite Solar Cells through In-Situ and Operando Electron Microscopy. Nano Energy 2018, 47, 243–256. 10.1016/j.nanoen.2018.02.055.

[ref41] Karlsson M.; Yi Z.; Reichert S.; Luo X.; Lin W.; Zhang Z.; Bao C.; Zhang R.; Bai S.; Zheng G.; Teng P.; Duan L.; Lu Y.; Zheng K.; Pullerits T.; Deibel C.; Xu W.; Friend R.; Gao F. Mixed Halide Perovskites for Spectrally Stable and High-Efficiency Blue Light-Emitting Diodes. Nat. Commun. 2021, 12 (1), 361 10.1038/s41467-020-20582-6. 33441549 PMC7806600

[ref42] Shikoh A. S.; Paek S.; Polyakov A. Y.; Smirnov N. B.; Shchemerov I. V.; Saranin D. S.; Didenko S. I.; Ahmad Z.; Touati F.; Nazeeruddin M. K. Assessing Mobile Ions Contributions to Admittance Spectra and Current-Voltage Characteristics of 3D and 2D/3D Perovskite Solar Cells. Sol. Energy Mater. Sol. Cells 2020, 215, 110670 10.1016/j.solmat.2020.110670.

[ref43] Mosconi E.; De Angelis F. Mobile Ions in Organohalide Perovskites: Interplay of Electronic Structure and Dynamics. ACS Energy Lett. 2016, 1 (1), 182–188. 10.1021/acsenergylett.6b00108.

[ref44] Ceratti D. R.; Zohar A.; Kozlov R.; Dong H.; Uraltsev G.; Girshevitz O.; Pinkas I.; Avram L.; Hodes G.; Cahen D. Eppur Si Muove: Proton Diffusion in Halide Perovskite Single Crystals. Adv. Mater. 2020, 32 (46), 2002467 10.1002/adma.202002467. 33048452

[ref45] Yang T.-Y.; Gregori G.; Pellet N.; Grätzel M.; Maier J. The Significance of Ion Conduction in a Hybrid Organic-Inorganic Lead-Iodide-Based Perovskite Photosensitizer. Angew. Chem. 2015, 127 (27), 8016–8021. 10.1002/ange.201500014. 25980541

[ref46] Mosconi E.; Meggiolaro D.; Snaith H. J.; Stranks S. D.; De Angelis F. Light-Induced Annihilation of Frenkel Defects in Organo-Lead Halide Perovskites. Energy Environ. Sci. 2016, 9 (10), 3180–3187. 10.1039/C6EE01504B.

[ref47] Amari S.; Verilhac J.-M.; Gros D’Aillon E.; Ibanez A.; Zaccaro J. Optimization of the Growth Conditions for High Quality CH3NH3PbBr3 Hybrid Perovskite Single Crystals. Cryst. Growth Des. 2020, 20 (3), 1665–1672. 10.1021/acs.cgd.9b01429.

[ref48] Kaltenbrunner M.; Adam G.; Głowacki E. D.; Drack M.; Schwödiauer R.; Leonat L.; Apaydin D. H.; Groiss H.; Scharber M. C.; White M. S.; Sariciftci N. S.; Bauer S. Flexible High Power-per-Weight Perovskite Solar Cells with Chromium Oxide–Metal Contacts for Improved Stability in Air. Nat. Mater. 2015, 14 (10), 1032–1039. 10.1038/nmat4388. 26301766

[ref49] Zhang Y.; Liu Y.; Xu Z.; Ye H.; Li Q.; Hu M.; Yang Z.; Liu S. Two-Dimensional (PEA)_2_PbBr_4_ Perovskite Single Crystals for a High Performance UV-Detector. J. Mater. Chem. C 2019, 7 (6), 1584–1591. 10.1039/C8TC06129G.

[ref50] Li J. V. Deep Level Transient Spectroscopy Characterization without the Arrhenius Plot. Rev. Sci. Instrum. 2021, 92 (2), 023902 10.1063/5.0039555. 33648155

