# Supplementary material for: Photoinduced Current Transient Spectroscopy on Metal Halide Perovskites: Electron Trapping and Ion Drift
Source: ACS Energy Lett. 2023 Sep 26;8(10):4371–9. doi: 10.1021/acsenergylett.3c01429 (PMC10580305; doi:10.1021/acsenergylett.3c01429)
Supplement: Supplementary file 1 — nz3c01429_si_001.pdf [file nz3c01429_si_001.pdf]

Supporting Information

Photoinduced Current Transient Spectroscopy on Metal Halide Perovskites: Electron Trapping and Ion Drift

Giovanni Armaroli,[#†] Lorenzo Maserati,*[#†] Andrea Ciavatti,[#] Pierpaolo Vecchi,[#] Alberto Piccioni,[#] Martina Foschi,[#] Valentina Van der Meer,[#] Chiara Cortese,[#] Matias Feldman,[#] Vito Foderà,[#] Thibault Lemercier,[§] Julien Zaccaro,[§] Javier Mayén Guillén,[‖] Eric Gros-Daillon,[‡] Beatrice Fraboni,[#] and Daniela Cavalcoli[#]

# Department of Physics and Astronomy, University of Bologna, 40127 Bologna, Italy.

§ University Grenoble Alpes, CNRS, Grenoble INP, Institut Néel, F38042 Grenoble, France.

‖ University Grenoble Alpes, CEA, Liten, F-38000 Grenoble, France.

‡ University Grenoble Alpes, CEA, Leti, F-38000 Grenoble, France.

† Equal contribution.

* Correspondence to: lorenzo.maserati@unibo.it

Table of contents

**Figure S1**	$(PEA)_2PbBr_4$ and $MAPbBr_3$ atomistic representation and samples' photographs
**Figure S2**	Photoinduced current transient spectroscopy (PICTS) simulations
**Figure S3**	Impedance spectroscopy (IS) on metal halide perovskites (MHPs): data and setup
**Figure S4**	Intensity modulated photocurrent spectroscopy (IMPS) on MHPs: data and setup
**Figure S5**	MHPs current-voltage (IV) plots over temperature
**Figure S6**	$MAPbBr_3$ current transients, highlighting reverse current spikes
**Figure S7**	Charge transport model for positive bias and experimental transients
**Figure S8**	$MAPbBr_3$ photocurrent transients – full dataset
**Figure S9**	Stability of the photocurrent transients
**Figure S10**	Repeatability of the PICTS measurements
**Figure S11**	PICTS measurements stress test and signal drift assessment
**Figure S12**	PICTS with coplanar electrodes configuration for $MAPbBr_3$
**Discussion S1**	Ion drift calculation from IS
**Discussion S2**	PICTS discussion
**Discussion S3**	Ion's assignment

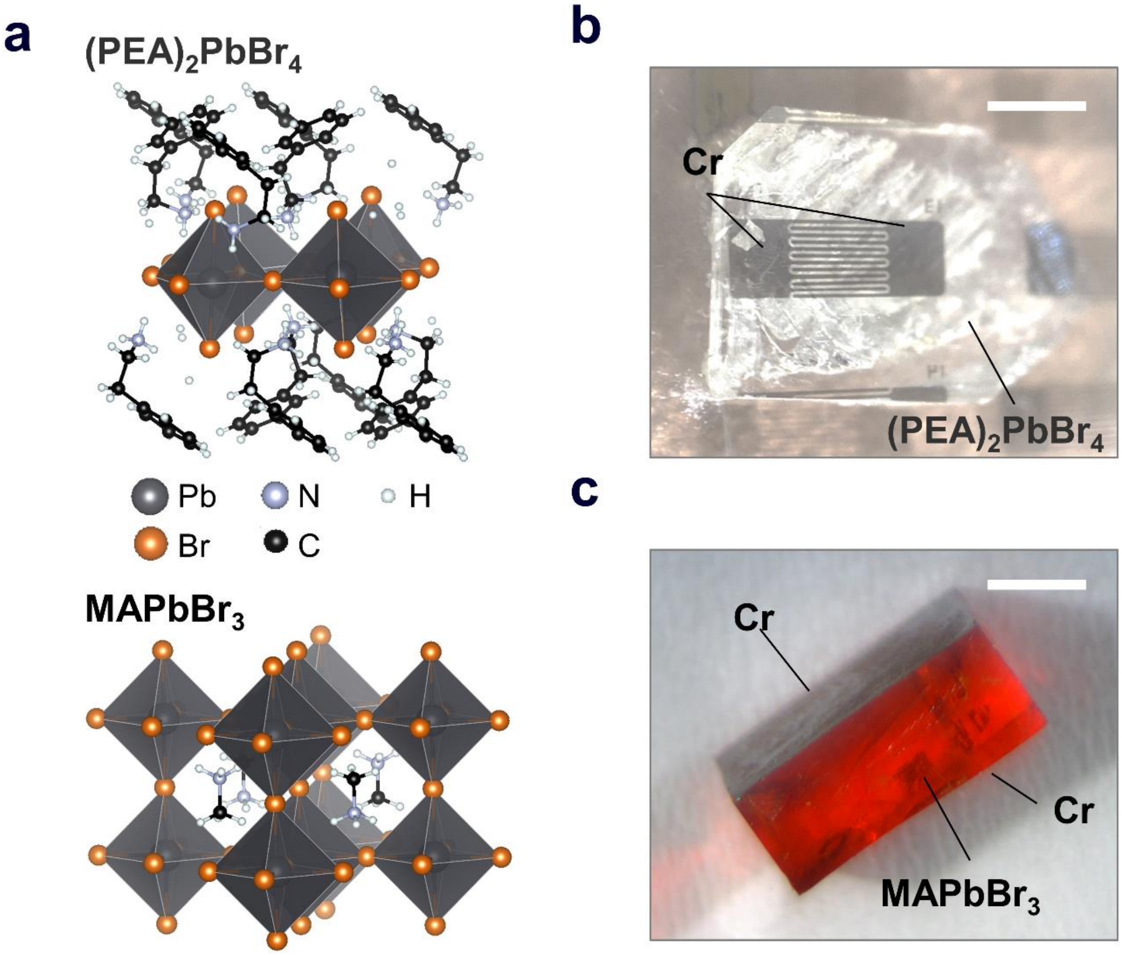

Figure S1. (a) Atomistic representation of metal bromine perovskites samples considered in this study: 2D-like $(PEA)_2PbBr_4$ (top) and $MAPbBr_3$ (bottom). **(b-c)** $(PEA)_2PbBr_4$ (b) and $MAPbBr_3$ (c) optical images of the samples with highlighted metal contacts. Scale bars, 1 mm.

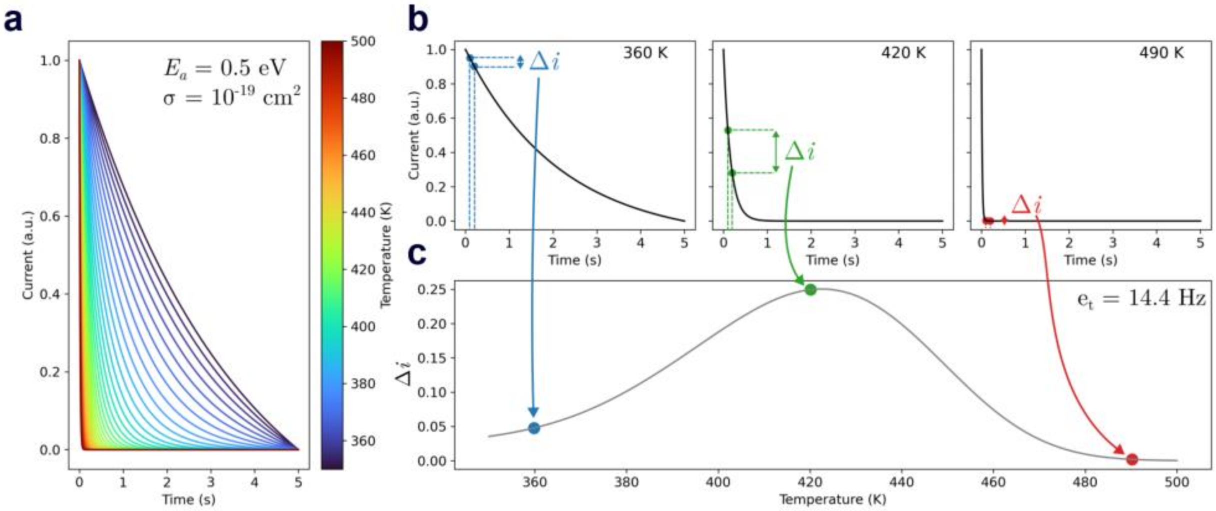

Figure S2. PICTS data analysis layout. **(a)** Simulated current transients for a single defect state with $E_a = 0.5\, eV$ and $\sigma = 10^{-19}\, cm^2$ in the $350 - 500\, K$ temperature range. Here E_a is the activation energy of the trapped charge carrier, σ is the trap's capture cross-section. For the complete description of the classic PITCS parameters see refs. [1,2]. **(b)** Selected transients from (a) at three different temperatures. For all three, the same rate window is selected, with $t_1 = 0.1\, s$ and $t_2 = 0.2\, s$. The corresponding Δi values are indicated by two-sided arrows. **(c)** The PICTS spectrum consists of a plot of the Δi values as a function of temperature. Coloured dots indicate the specific Δi values calculated from the transients in (b). This spectrum is related to the rate window $e_t = 14.4\ \mathrm{Hz}$, determined by the t_1 and t_2 values.

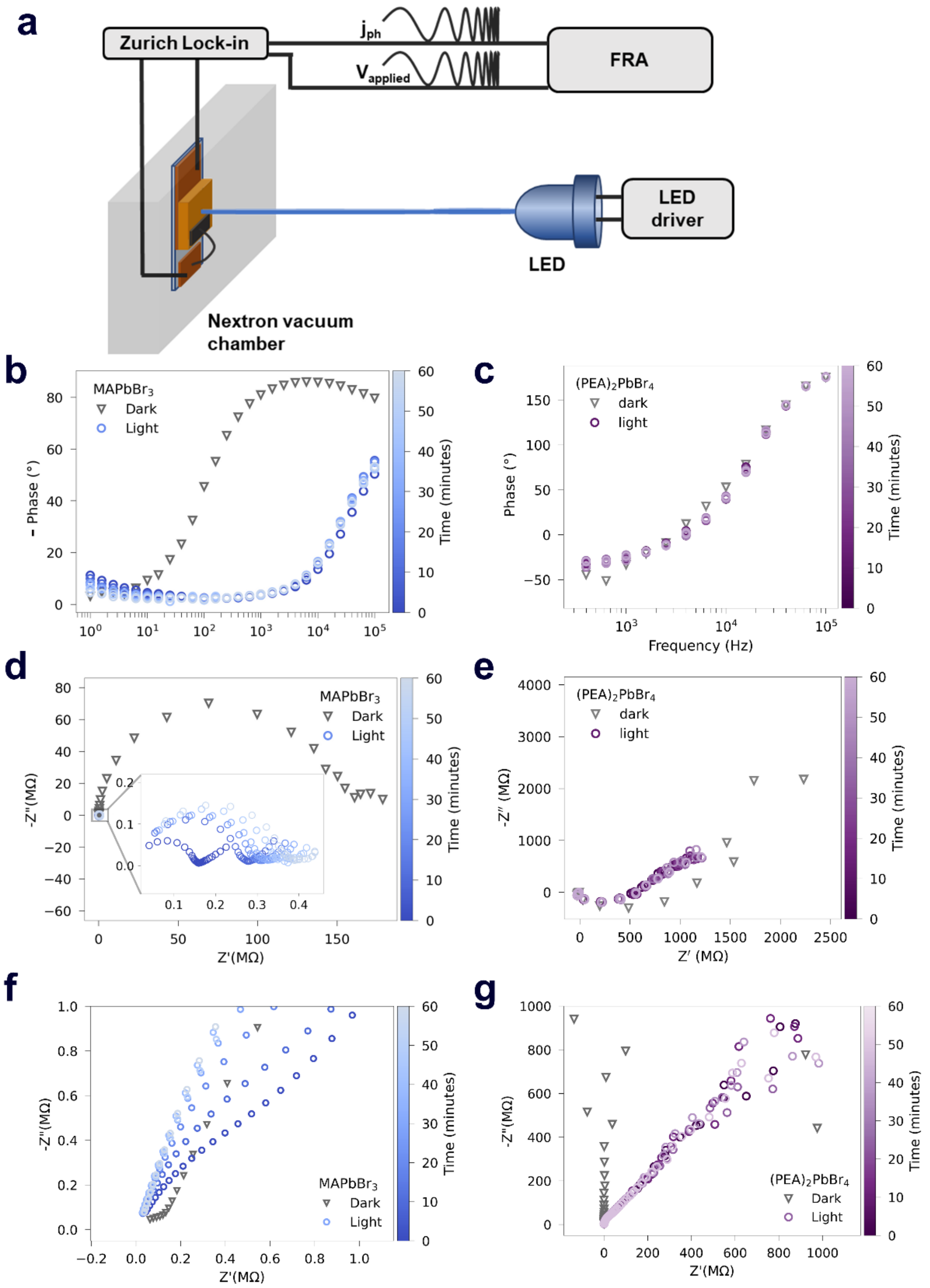

Figure S3. Impedance spectroscopy (IS) extended data. **(a)** IS experimental setup: a LED driver connected to either a $470\ nm$ or $365\ nm$ LED (for $MAPbBr_3$ and $(PEA)_2PbBr_4$ respectively) shines light onto the sample, inducing a photocurrent. The sample is polarized with a $+5\ V$ external bias applied through a lock-in amplifier. The frequency response analyzer (FRA) module of the lock-in amplifier was used to induce an AC voltage perturbation of amplitude $200\ mV$, with frequency

varying from $100\,kHz$ down to $1\,Hz$. The impedance corresponding to each frequency was retrieved through the FRA as the ratio between perturbation voltage and current signal Fourier component with same frequency of the voltage perturbation, whose intensity and phase are extracted by the lock-in amplifier. **(b-c)** IS phase plots corresponding to the intensity plots reported in Figure 2a-b, main text, for $MAPbBr_3$ and $(PEA)_2PbBr_4$ respectively. The data were acquired in the dark first and then under illumination (intensity $5\,mW/cm^2$), repeating the IS measurement every 3 min to acquire the time evolution. **(d-e)** Corresponding Nyquist plots, for $MAPbBr_3$ (d) and $(PEA)_2PbBr_4$ (e), respectively. Comparing the two plots, $MAPbBr_3$ signal clearly displayed a more relevant signal evolution over time. **(f-g)** Nyquist plots obtained setting the bias at $0\,V$ to demonstrate the influence of the sole illumination on the sample impedance over time. Again, in the $MAPbBr_3$ case (f), the signal clearly shows a more significant time evolution compared to the $(PEA)_2PbBr_4$ case (g).

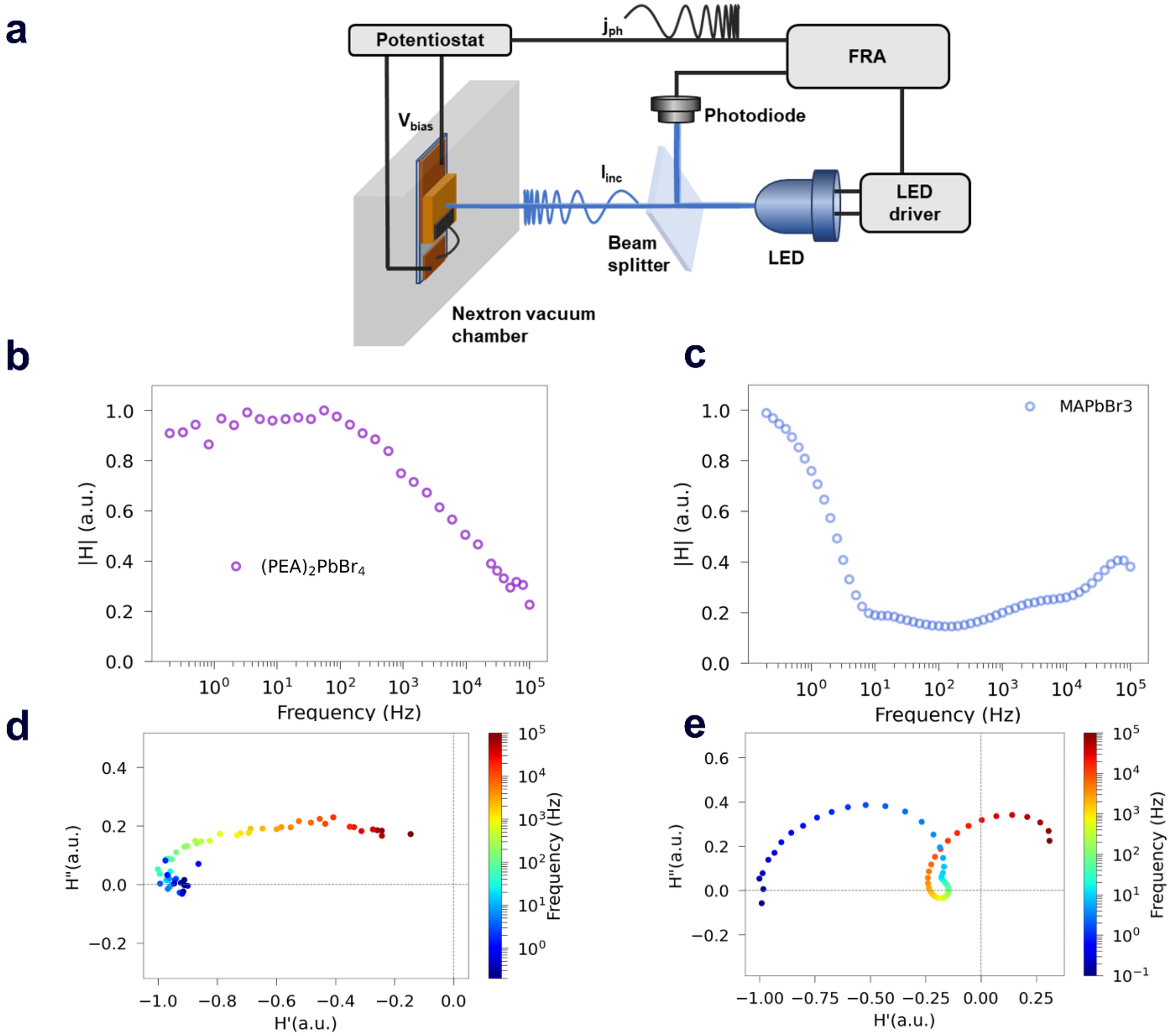

Figure S4. Intensity modulated photocurrent spectroscopy (IMPS) extended data. **(a)** IMPS setup schematic. The sample is mounted in a vacuum chamber (Nextron) and electrically connected to

an external circuit. A frequency response analyzer (FRA) module records the sample photocurrent and at the same time, the incident light intensity (*via* a photodiode). The FRA also controls the LED driver connected to either a $470\,nm$ or $365\,nm$ LED (for $MAPbBr_3$ and $(PEA)_2PbBr_4$ respectively), driven at a sweeping with frequency varying from $100\ kHz$ down to $0.1\ Hz$. The light is divided by a beam splitter to have 50% illumination on the sample and 50% illumination on the photodiode. The FRA module is controlled by a potentiostat. **(b-c)** IMPS intensity plots corresponding to the phase plots of Figure 2c, main text for the 2D (b) and 3D (c) perovskite, respectively. **(d-e)** IMPS Nyquist plots of the $(PEA)_2PbBr_4$ (d) and $MAPbBr_3$ (e), respectively.

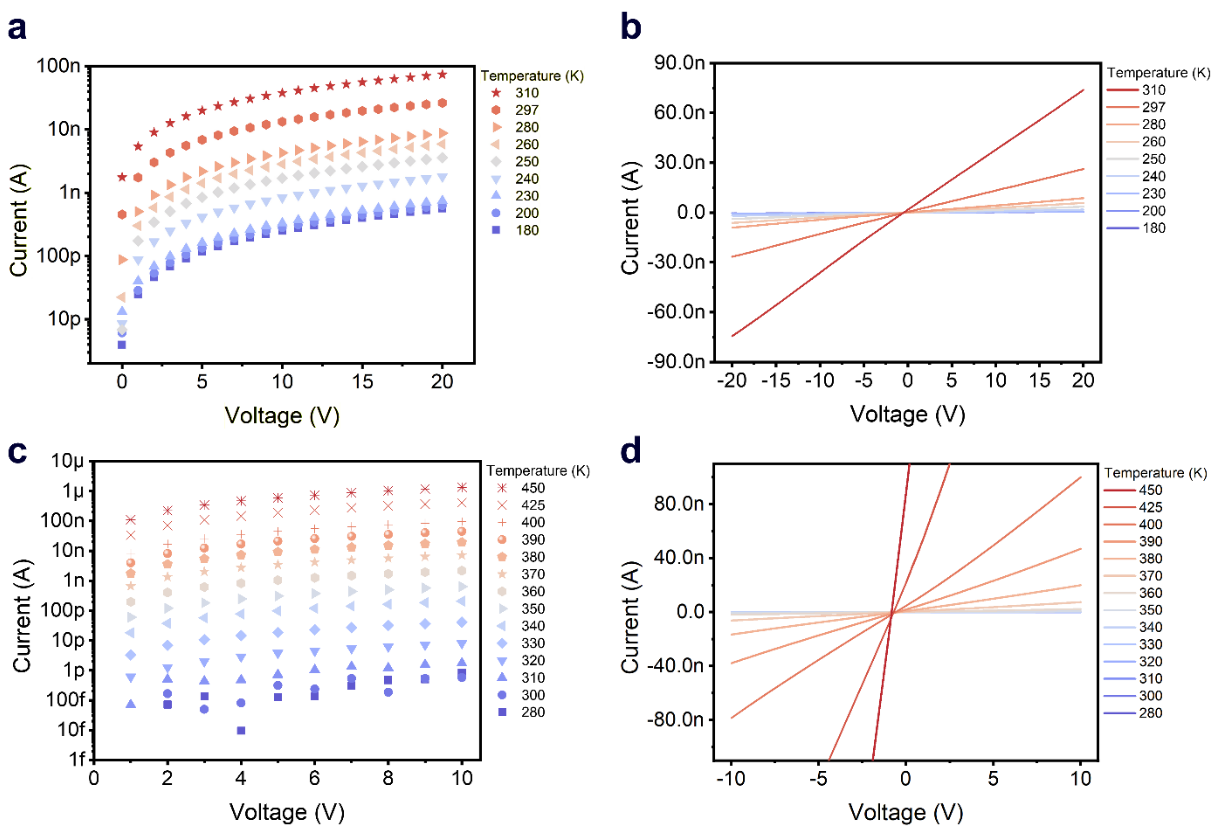

Figure S5. $(PEA)_2PbBr_4$ (**a,b**) and $MAPbBr_3$ (**c,d**) current versus voltage plots over temperature in logarithmic scale (a,c) and linear scale (b,d) used to calculate the ion conductivity activation energy, and showing Ohmic behavior.

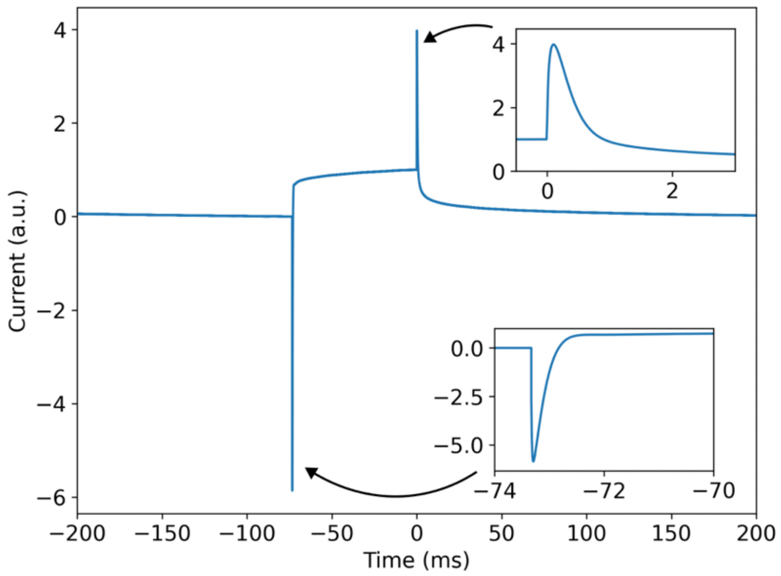

Figure S6. $MAPbBr_3$ photocurrent transient measured at a $70\,ms$ excitation time. The insets highlight the reverse current spikes when the light is turned on (first spike from the left) and turned off (second spike from the left). Bias voltage $-5\,V$, illumination $470\,nm$, $5\,mW/cm^2$.

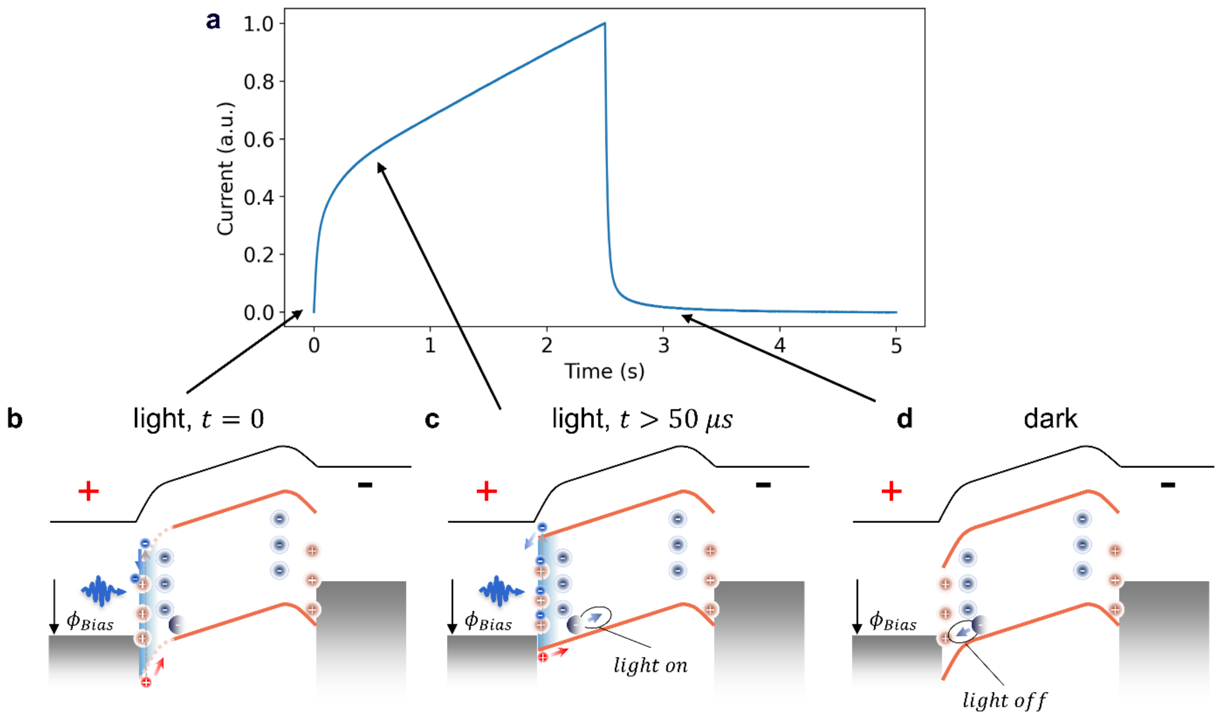

Figure S7. (a) Normalized photocurrent transient at room temperature for $+5\,V$ bias and illumination pulse duration $2.5\,s$. **(b-d)** Band diagram models for illustrating the effect of intermittent illumination over time at the semitransparent electrode, where a positive bias is applied.

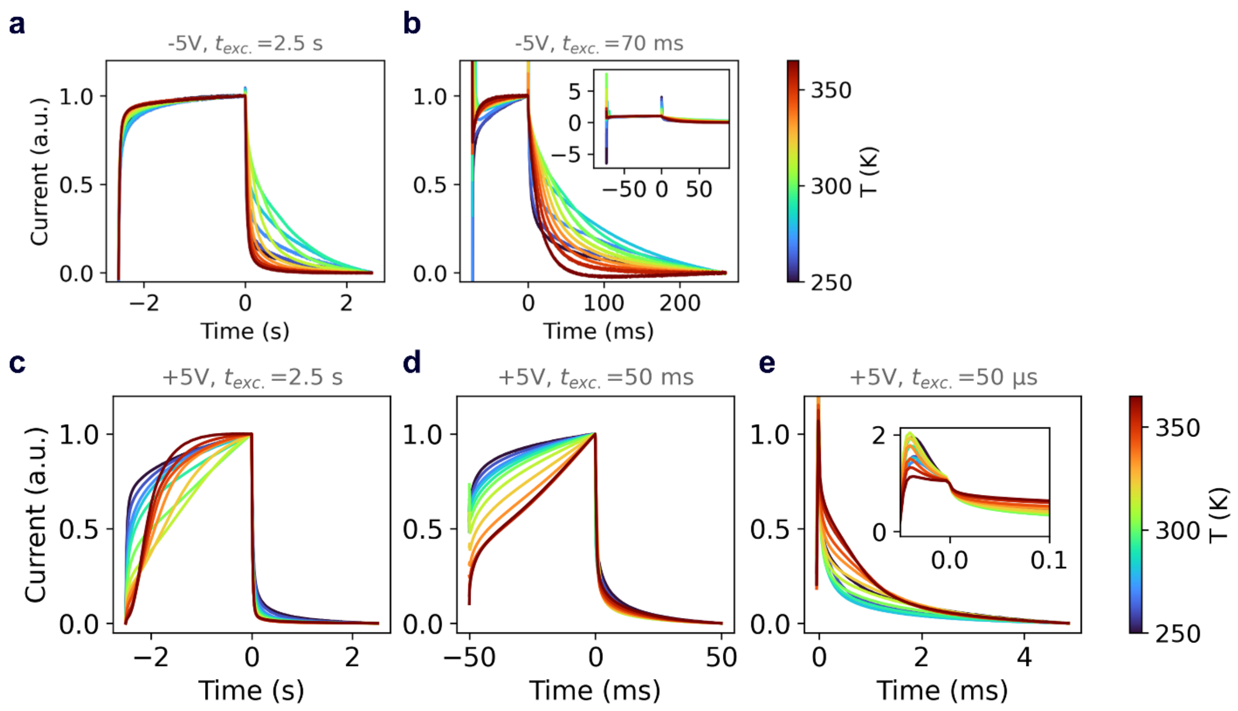

Figure S8. Complete photocurrent transient dataset used to derive the Arrhenius plots reported in main text Figure 4e. Positive (**a-b**) and negative (**c-e**) bias and slow (a,c) medium, (b,d) and high (e) light modulation frequencies are used to distinguish ionic charge sign and ion species, respectively. Note: no readable signal was observed for positive bias and high frequency.

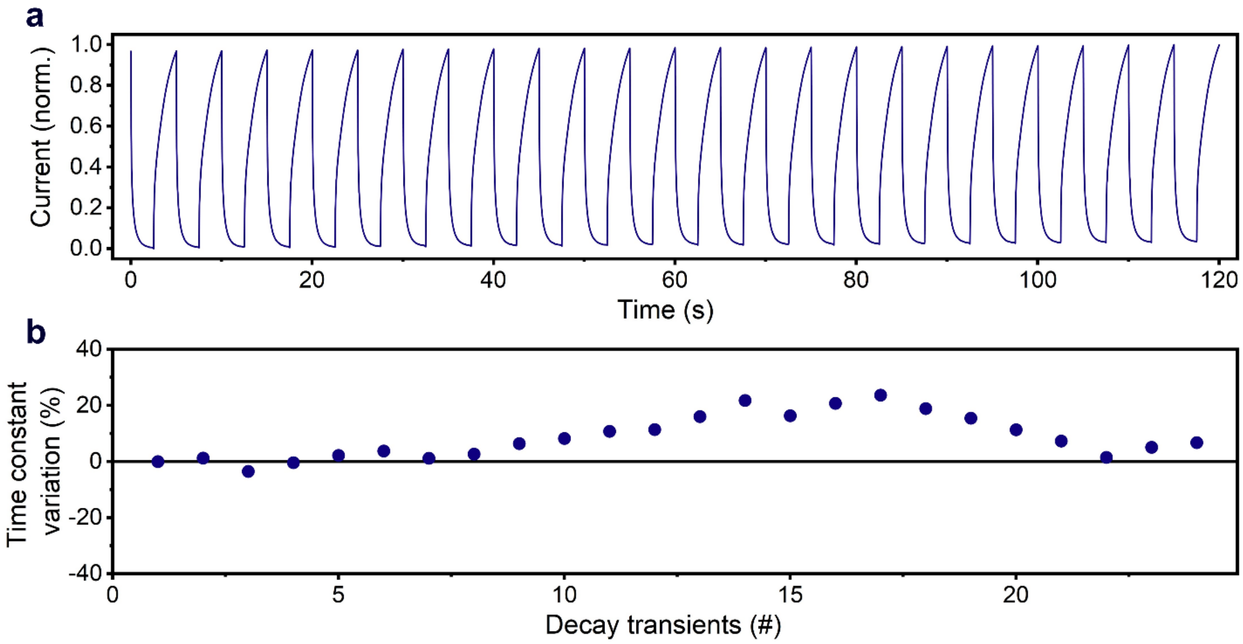

Figure S9. Stability of the photocurrent transients used to carry out PICTS analysis. The $MAPbBr_3$ sample was loaded in the cryostat at room temperature in vacuum and the current flowing through

its contacts recorded over time, modulated by intermittent blue light illumination ($470\,nm$, $5\,mW/cm^2$, period $2.5\,s$) keeping the voltage bias fixed at $+5\,V$, and temperature fixed at $300\,K$. **(a)** Normalized current versus time. **(b)** Dominant time constant variation of the current decays reported in (a). To extract the time constant each current decay was fitted with a double exponential decay and the slowest time constant (around $0.4\,s$) considered. The time constants were subsequently normalized against the initial value to show their percentage variation over time.

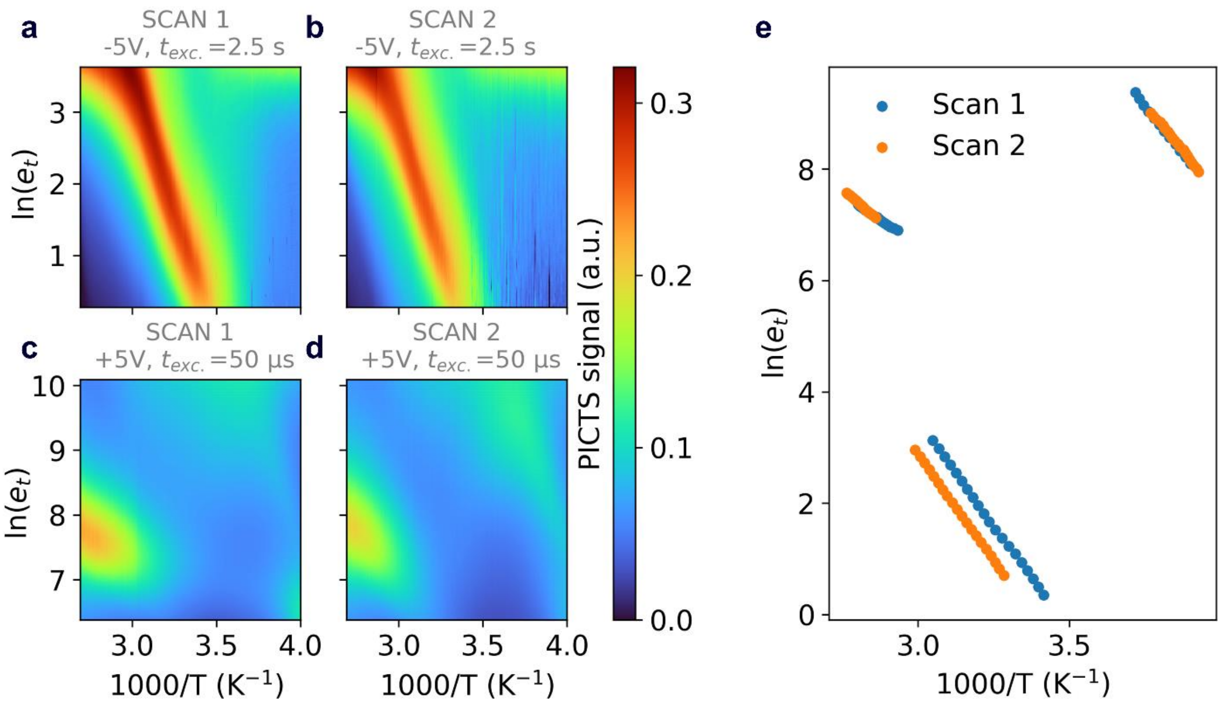

Figure S10. PICTS measurements repeatability test. **(a-d)** PICTS maps corresponding to measurements carried out at different voltages: $-5\,V$ (a,b), $+5\,V$ (c,d), at subsequent times, *i.e.* measurements reported in (a,c) preceded the measurements (c,d) carried out on the same sample. **e,** Arrhenius plots of the maxima displayed in (a,b,c,d) in red versus inverse temperature showing good repeatability.

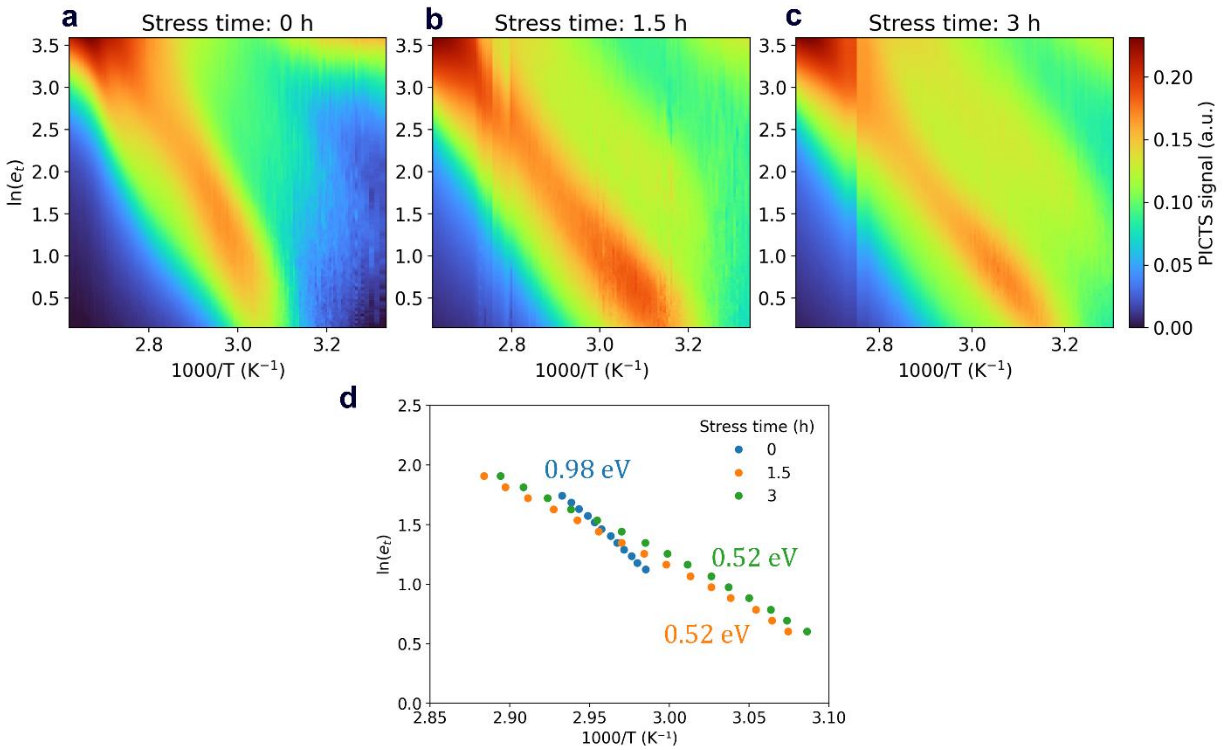

Figure S11. PICTS measurements stress test and signal drift assessment under vacuum, applied external bias and intermittent illumination over a time span of hours. **(a-c)** PICTS maps corresponding to measurements carried out at different times: $t = 0\ h$ (a), $t = 1.5\ h$ (b), and $t = 3\ h$ (c). **(d)** Arrhenius plots of maxima displayed in (a,b,c) in red versus inverse temperature, showing a good signal reproducibility.

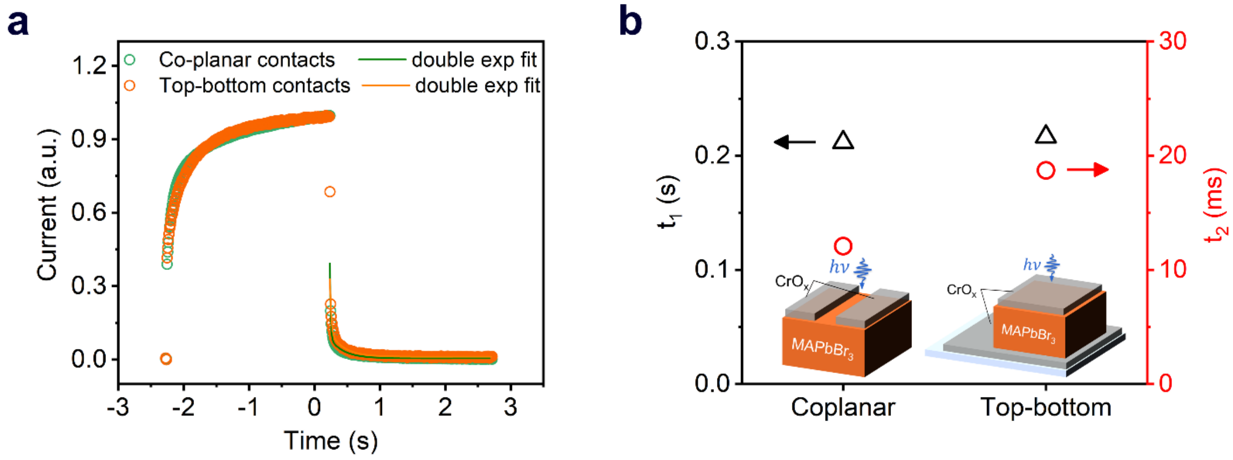

Figure S12. Comparison of photocurrent transients for the coplanar and the top-bottom contacts architecture on the $MAPbBr_3$ single crystal. **(a)** Normalized photocurrent transients for the coplanar (green) and the top-bottom (orange) contacts, scattered dots, fitted in the photocurrent decay region with double exponential decays (lines). **(b)** Time constants of the decays retrieved from the fitting of data displayed in (a) demonstrating very similar decay behavior.

Supporting Discussion

Ion drift calculation from impedance spectroscopy (IS)

Based on the experimental data reported in the main text Figure 2a,b, we derived the following calculations for describing the time evolution of the sample impedance, correlating the value to the ion diffusivity. We argue that the photoinduced mobile ions slowly drift across the entire crystal of thickness W, under the external potential bias ($+5\,V$) applied such that: $W = 1\,mm$; $E = V/W = 5\,V/mm$. Therefore, the ion diffuses according to the Einstein–Smoluchowski equation:

$$D = \mu \frac{k_B T}{q} = \frac{v}{E}\frac{k_B T}{q} = \frac{W}{\tau}\frac{1}{E}\frac{k_B T}{q}$$

Here D is the ion diffusion coefficient, μ is the ion mobility, k_B is the Boltzmann constant, T is the temperature, q is the elementary charge. The ion mobility μ can be rewritten as drift velocity v_D over the electric field E imposed by an external bias. The drift velocity can be further decomposed considering the crystal thickness separating the electrodes W and the transient time τ. While all the other parameters' values are known, we retrieved the transient time value of $\tau = 3000 \pm 1500\,s$ by the exponential fitting displayed in Figure 2a inset, main text. By plugging in the values, we get:

$$D = \frac{1\,mm}{3000\,s}\left(\frac{5\,V}{mm}\right)^{-1} 25\,mV = \frac{1\,mm^2}{3000\,s}\left(\frac{25\,mV}{5\,V}\right) = \frac{(10^{-1}cm)^2}{3000\,s}\left(\frac{0.025\,V}{5\,V}\right) = \frac{5 \times 10^{-5}\,\mathrm{cm}^2}{3000\,s} =$$
$$= 1.7 \times 10^{-8}\,\mathrm{cm}^2/s$$

Therefore, due the external bias and continuous illumination, ions drift across the entire crystal with a high time constant, that corresponds to an average diffusivity value for all the ionic species falling in the $10^{-8}\,\mathrm{cm}^2/\mathrm{s}$ range.

PICTS Discussion

In addition to the electric field imposed by the external bias, light absorption at the MHP surface causes a transient variation of the fixed charges screening, thus a modification of an additional local electric filed. This, in turn, causes a local drift of ionic species. Thanks to different light modulation frequencies we can select different ionic species that move at different speeds, or in other terms, ions having different diffusion coefficients (due to the link between mobility and diffusion).

From the impedance spectroscopy measurements under illumination we obtained an impedance evolving with time constant $\tau{\sim}3000\,s$, in line with reported values in literature,[3,4] which is orders of magnitude higher than the maximum probed time of $2.5\,s$ in our photocurrent transients. This means that during the single PICTS transients, the overall ionic current flowing from one metal contact to another is not affecting the extracted photocurrent. In fact, by considering the longest $2.5\,s$ light pulse (Figure S8), we can calculate an upper limit for the ion drift length (in addition to the already drifting ions under bias in dark condition) of $\sim 30\,nm$.[5] The spatial extend of such drift does not significantly alter the sample global ion distribution during the

single measurement time-span (see stability and repeatability checks in Figures S9, S10) , but it does induce a reversible ion accumulation on the hour time-scale if the bias voltage (and/or illumination) is held constant (Figure S11). The bias voltage we set was chosen to maximize the current without excessively affecting the baseline. The architectural configuration of the top-bottom contacts had no influence on the value of the photocurrent transient time constants as demonstrated by a comparison with photocurrent transients obtained from a coplanar architecture reported in Figure S12.

The ion drift upon light switching is analogous to the ion drift in a depletion region captured by transient-ion-drift (TID) measurements, which have been extensively carried out on MHPs.[6–9] Such motion induces a transient impedance change of the form $Z = Z_0 \exp\left(-\frac{t}{\tau}\right)$, where Z_0 is the space charge region (SCR) impedance when light is turned off, and τ is the characteristic time of ionic motion. Considering the relation $v_{drift} = \mu E$, and the Einstein–Smoluchowski equation for the diffusion of charged particles ($D = \mu k_B T/q$) in the depletion layer, the migration rate of ions during the drift process can be written as:[6,7,10]

$$e_t(T) = \frac{1}{\tau(T)} = \frac{v_{drift}}{W_D} = \frac{qE}{W_D k_B T} D(T) = \frac{qE}{W_D k_B T} D_0 \exp\left(-\frac{E_a}{k_B T}\right) \quad (1)$$

where T is the temperature, W_D is the depletion layer (or space charge region) width, q is the elementary charge, E is the electric field in the SCR, k_B is the Boltzmann constant, D_0 is the ion diffusion coefficient at $T = \infty$, and E_a is the diffusion activation energy. The change in the ionic impedance at the contact has a direct influence on the electronic motion in the material. Therefore, the ion migration rate is found also in the expression of the electronic photocurrent decay:

$$i(t) = i_{dark} + i_0 \exp(-e_t \cdot t) \quad (2)$$

where i_{dark} is the dark current value, i_0 is the photocurrent amplitude at steady state, and e_t is the ion migration rate from Eq. (1). The latter describes an Arrhenius-type dependence of e_t as a function of temperature, which resembles the one obtained in classical PICTS due to emission of charge carriers from deep traps. Because of this, PICTS signals in MHPs arising from ionic motion can easily be misinterpreted as coming from deep electronic states. Therefore, determining if ions play a major role the photocurrent transients become pivotal for discriminating two different physical processes that lead to a description of different quantities using PICTS experiments: trap state energies or ion diffusion activation energies.

Table 1 in the main text, reports the activation energies and diffusion coefficients at room temperature obtained by a linear fitting of the Arrhenius plots in Figure 4e by Eq. (1). While the activation energy is easily obtainable by the fit slope, the diffusion coefficient requires the knowledge of E and W_D which are parameters difficult to assess experimentally. Thus, the reported D_{300K} values are a rough estimate, based on the values of $E = 0.3\ V/\mu m$, and $W_D = 1\ \mu m$. The former is based on the value measured by kelvin probe force microscopy.[11] The latter is derived from values reported in ref. [11] and ref. [12], where the band bending at the MHP−metal interface for both $MAPbI_3$, and $MAPbBr_3$ is characterized to be in the microns range and we

consider a conservative estimate based on the partial compensation of the interfacial charges responsible for the SCR by the photogenerated charge carriers.

Ion's assignment

We provide a tentative assignment of the reported PICTS maxima (traces) to migrating ionic species in $MAPbBr_3$, that are reported in Table 1, main text. We assign the traces measured with negative bias to positively charged ions, and features measured with positive bias to negatively charged ones. Therefore, the traces reported in Figure 4, main text, LN and MN should correspond to positively charged species. The most mobile charged ionic species in MHPs are $\mathrm{MA}_i^{\bullet}$, and $\mathrm{V}_{\mathrm{Br}}^{\bullet}$, *i.e.* methylammonium interstitials and bromine vacancies, while $\mathrm{Pb}_{\mathrm{i}}^{\bullet\bullet}$ is expected to be rather immobile in the lattice.[13] Reichert *et al.* assigned the γ trace to $\mathrm{MA}_i^{\bullet}$,[7] and, given the good overlap of LN with γ, we assign LN to this ionic species. We associate the higher activation energy that we measured with respect to the one reported for γ to the absence of grain boundaries in the single crystal samples. By exclusion, we assign MN to $\mathrm{V}_{\mathrm{Br}}^{\bullet}$ migration. Given the vicinity of MP and HP1 in the Arrhenius space, we assign them to the same negatively charged ionic species. Given the good overlap with the δ trace reported in ref. [7], which was assigned to halide interstitials,[7] we assign them to $\mathrm{Br}_{\mathrm{i}}'$ migration. The difference in activation energy of around 0.2 eV between MP and HP1 might be related to the presence of the second HP2 trace in the high frequency PICTS map. Indeed, the presence of several features in the spectra can lead to convolutions between the peaks, that mask the real activation energy of the underlying phenomena. The HP2 feature shows a good overlap with the β trace,[7] which was assigned to $\mathrm{V}_{\mathrm{MA}}'$, in good agreement with the negative charge expected for this defect. However, as discussed above, the estimated diffusion coefficient of HP2 is not compatible with an ionic migrating species. Therefore, this trace needs further investigation to assess its nature. It is important to note the difficulty of assigning traces from defect spectroscopy to the underlying ionic migrating species. This task has proven to be difficult not only by the experimental challenges of these measurements, but also to the intrinsically complex nature of ion migration in MHPs. As an example, as discussed above the β trace was assigned to $\mathrm{V}_{\mathrm{MA}}'$,[7] but recently the same group reassigned it to $\mathrm{V}_{\mathrm{I}}^{\bullet}$,[14] thus changing not only the moving chemical specie, but also its charge. However, β was also observed by the same group in MHPs without iodine, like $CsFAPbBr_{1-x}Cl_x$,[15] and, on the other hand, it was *not* observed in a iodine-based MHP like $FAPbI_3$.[16] This exemplifies how difficult is the task of assigning defect spectroscopy traces to their related ionic species. Therefore, we note that the assignments reported above should be taken with caution and may be changed in the future upon further investigation.

References

(1) Balland, J. C.; Zielinger, J. P.; Noguet, C.; Tapiero, M. Investigation of Deep Levels in High-Resistivity Bulk Materials by Photo-Induced Current Transient Spectroscopy. I. Review and Analysis of Some Basic Problems. *J. Phys. Appl. Phys.* **1986**, *19* (1), 57–70. https://doi.org/10.1088/0022-3727/19/1/011.

(2) Balland, J. C.; Zielinger, J. P.; Tapiero, M.; Gross, J. G.; Noguet, C. Investigation of Deep Levels in High-Resistivity Bulk Materials by Photo-Induced Current Transient Spectroscopy. II. Evaluation of Various Signal Processing Methods. *J. Phys. Appl. Phys.* **1986**, *19* (1), 71–87. https://doi.org/10.1088/0022-3727/19/1/012.

(3) García-Batlle, M.; Baussens, O.; Amari, S.; Zaccaro, J.; Gros-Daillon, E.; Verilhac, J.; Guerrero, A.; Garcia-Belmonte, G. Moving Ions Vary Electronic Conductivity in Lead Bromide Perovskite Single Crystals through Dynamic Doping. *Adv. Electron. Mater.* **2020**, *6* (10), 2000485. https://doi.org/10.1002/aelm.202000485.

(4) Ceratti, D. R.; Zohar, A.; Kozlov, R.; Dong, H.; Uraltsev, G.; Girshevitz, O.; Pinkas, I.; Avram, L.; Hodes, G.; Cahen, D. Eppur Si Muove: Proton Diffusion in Halide Perovskite Single Crystals. *Adv. Mater.* **2020**, *32* (46), 2002467. https://doi.org/10.1002/adma.202002467.

(5) Eames, C.; Frost, J. M.; Barnes, P. R. F.; O'Regan, B. C.; Walsh, A.; Islam, M. S. Ionic Transport in Hybrid Lead Iodide Perovskite Solar Cells. *Nat. Commun.* **2015**, *6* (May), 2–9. https://doi.org/10.1038/ncomms8497.

(6) Futscher, M. H.; Lee, J. M.; McGovern, L.; Muscarella, L. A.; Wang, T.; Haider, M. I.; Fakharuddin, A.; Schmidt-Mende, L.; Ehrler, B. Quantification of Ion Migration in $CH_3NH_3PbI_3$ Perovskite Solar Cells by Transient Capacitance Measurements. *Mater. Horiz.* **2019**, *6* (7), 1497–1503. https://doi.org/10.1039/c9mh00445a.

(7) Reichert, S.; An, Q.; Woo, Y. W.; Walsh, A.; Vaynzof, Y.; Deibel, C. Probing the Ionic Defect Landscape in Halide Perovskite Solar Cells. *Nat. Commun.* **2020**, *11* (1). https://doi.org/10.1038/s41467-020-19769-8.

(8) Li, B.; Kan, C.; Hang, P.; Fang, Y.; Zuo, L.; Song, L.; Zhang, Y.; Yang, D.; Yu, X. Understanding the Influence of Cation and Anion Migration on Mixed-Composition Perovskite Solar Cells via Transient Ion Drift. *Phys. Status Solidi RRL – Rapid Res. Lett.* **2021**, *15* (9), 2100225. https://doi.org/10.1002/pssr.202100225.

(9) Urbaniak, A.; Czudek, A.; Dagar, J.; Unger, E. L. Capacitance Spectroscopy of Thin-Film Formamidinium Lead Iodide Based Perovskite Solar Cells. *Sol. Energy Mater. Sol. Cells* **2022**, *238*, 111618. https://doi.org/10.1016/j.solmat.2022.111618.

(10) Heiser, T.; Mesli, A. Determination of the Copper Diffusion Coefficient in Silicon from Transient Ion-Drift. *Appl. Phys. A* **1993**, *57* (4), 325–328. https://doi.org/10.1007/BF00332285.

(11) Ahmadi, M.; Collins, L.; Higgins, K.; Kim, D.; Lukosi, E.; Kalinin, S. V. Spatially Resolved Carrier Dynamics at $MAPbBr_3$ Single Crystal–Electrode Interface. *ACS Appl. Mater. Interfaces* **2019**, *11* (44), 41551–41560. https://doi.org/10.1021/acsami.9b16287.

(12) Shrestha, S.; Tsai, H.; Yoho, M.; Ghosh, D.; Liu, F.; Lei, Y.; Tisdale, J.; Baldwin, J.; Xu, S.; Neukirch, A. J.; Tretiak, S.; Vo, D.; Nie, W. Role of the Metal–Semiconductor Interface in Halide Perovskite Devices for Radiation Photon Counting. *ACS Appl. Mater. Interfaces* **2020**, *12* (40), 45533–45540. https://doi.org/10.1021/acsami.0c11805.

(13) Mosconi, E.; De Angelis, F. Mobile Ions in Organohalide Perovskites: Interplay of Electronic Structure and Dynamics. *ACS Energy Lett.* **2016**, *1* (1), 182–188. https://doi.org/10.1021/acsenergylett.6b00108.

(14) Tammireddy, S.; Reichert, S.; An, Q.; Taylor, A. D.; Ji, R.; Paulus, F.; Vaynzof, Y.; Deibel, C. Temperature-Dependent Ionic Conductivity and Properties of Iodine-Related Defects in Metal Halide Perovskites. *ACS Energy Lett.* **2022**, *7* (1), 310–319. https://doi.org/10.1021/acsenergylett.1c02179.

(15) Karlsson, M.; Yi, Z.; Reichert, S.; Luo, X.; Lin, W.; Zhang, Z.; Bao, C.; Zhang, R.; Bai, S.; Zheng, G.; Teng, P.; Duan, L.; Lu, Y.; Zheng, K.; Pullerits, T.; Deibel, C.; Xu, W.; Friend, R.; Gao, F. Mixed Halide Perovskites for Spectrally Stable and High-Efficiency Blue Light-Emitting Diodes. *Nat. Commun.* **2021**, *12* (1), 361. https://doi.org/10.1038/s41467-020-20582-6.

(16) Teng, P.; Reichert, S.; Xu, W.; Yang, S.-C.; Fu, F.; Zou, Y.; Yin, C.; Bao, C.; Karlsson, M.; Liu, X.; Qin, J.; Yu, T.; Tress, W.; Yang, Y.; Sun, B.; Deibel, C.; Gao, F. Degradation and Self-Repairing in Perovskite Light-Emitting Diodes. *Matter* **2021**, *4* (11), 3710–3724. https://doi.org/10.1016/j.matt.2021.09.007.
